# Benchmarking the utility of maps of dynamics for human-aware motion planning

**DOI:** 10.3389/frobt.2022.916153

**Published:** 2022-11-02

**Authors:** Chittaranjan Srinivas Swaminathan, Tomasz Piotr Kucner, Martin Magnusson, Luigi Palmieri, Sergi Molina, Anna Mannucci, Federico Pecora, Achim J. Lilienthal

**Affiliations:** ^1^ AASS Lab, School of Science and Technology, Örebro University, Örebro, Sweden; ^2^ Finnish Centre for Artificial Intelligence (FCAI), Department of Electrical Engineering and Automation, Aalto University, Espoo, Finland; ^3^ Robert Bosch GmbH Corporate Research, Stuttgart, Germany; ^4^ Lincoln Centre for Autonomous Systems, School of Computer Science, University of Lincoln, Lincoln, United Kingdom

**Keywords:** human-aware motion planning, maps of dynamics, dynamic environments, benchmarking, human-populated environments, ATC

## Abstract

Robots operating with humans in highly dynamic environments need not only *react* to moving persons and objects but also to *anticipate and adhere to* patterns of motion of dynamic agents in their environment. Currently, robotic systems use information about dynamics locally, through tracking and predicting motion within their direct perceptual range. This limits robots to reactive response to observed motion and to short-term predictions in their immediate vicinity. In this paper, we explore how *maps of dynamics* (MoDs) that provide information about motion patterns outside of the direct perceptual range of the robot can be used in motion planning to improve the behaviour of a robot in a dynamic environment. We formulate cost functions for four MoD representations to be used in any optimizing motion planning framework. Further, to evaluate the performance gain through using MoDs in motion planning, we design objective metrics, and we introduce a simulation framework for rapid benchmarking. We find that planners that utilize MoDs waste less time waiting for pedestrians, compared to planners that use geometric information alone. In particular, planners utilizing both intensity (proportion of observations at a grid cell where a dynamic entity was detected) and direction information have better task execution efficiency.

## 1 Introduction

**FIGURE 1:**
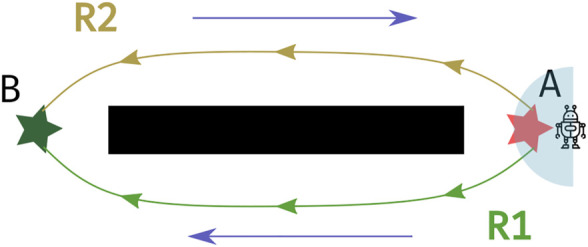
Two corridors on either side of a large central obstacle (black wall). Blue arrows indicate the typical direction of motion of dynamic entities. R1 and R2 are possible paths of equal length that can be taken by a robot to go from A (red star) to B (green star). When equipped with information about the typical patterns of motion *via* a Map of Dynamics (MoD), the robot will choose R1, the path that conforms with the blue arrows.

What motion-planning can gain from being aware of patterns of motion in its environment can be seen in the scenario visualized in Figure 1: two corridors separated by a wall. Suppose that entities (e.g., humans or human-driven vehicles) move in the environment along the directions indicated by the blue arrows, that is, predominantly towards the left in the bottom corridor and predominantly towards the right in the top corridor. Now, a robot operating alongside these dynamic entities has to move from A to B. Typically, a motion planning algorithm would return the shortest obstacle-free path from A to B, which, in this symmetric environment, could be R1 or R2 along either side of the corridor. However, in dynamic environments, robots should also account for patterns of motion of dynamic agents in their environment. This can be seen as follows: with standard motion planning approaches the robot will follow the path computed by a global planer (accounting only for static objects in the environment) and locally react to moving entities observed within its sensing range (light blue semi-circle shown in Figure 1). However, if the robot adopts path R2, which is against the direction in which entities tend to move in the respective area, it will more likely encounter approaching entities and thus need to stop, re-plan or maneuver around them more often. In contrast, if the robot adopts path R1, it will move along the same direction in which the other entities tend to move thus minimizing the number of avoidance maneuvers or stops. Notice that using information from what the robot can immediately observe is not enough to ensure that the motion planner chooses path R1. Therefore, information about the general patterns of dynamics in an environment is beneficial, if not necessary, for efficient robot operation.

From the point of view of motion planning, two types of information about dynamics can be useful: dynamics information associated with an entity and dynamics information associated with the environment. Information associated with dynamic entities is usually available live as positions of tracked entities or as the output of a motion prediction pipeline. Using motion prediction, the positions of tracked entities can be extrapolated into the future and can be used to re-plan robot paths to avoid it colliding with the entities (Rudenko et al., 2020). Information associated with the environment captures the general patterns of motion in the environment. Such information is referred to as *Maps of Dynamics* or MoDs for short (Kucner et al., 2020b). MoDs are spatially organized and can model motion patterns over the spatial domain. In this paper, we focus on using MoDs in motion planning and not live dynamics information.

Motion planning is often performed hierarchically and split into two phases: global and local (see the work by Lu et al. (2014)). *Global planning* is the phase of motion planning that generally happens before the robot starts moving. It constitutes a general plan (usually a sequence of motions to execute or poses to reach) that a robot should use to reach the goal pose from its current pose, taking into account the obstacles in a global cost map. When MoDs are used in the global planning phase, it is possible to plan motions that also account for the general patterns of dynamics represented with MoDs. *Local planning*, on the other hand, utilizes information immediately surrounding the robot (the local cost map created using sensory information) and tries to steer the robot along the global plan, as closely as possible. MoDs particularly benefit global planning, since information over the entire spatial domain can be available: it is analogous to adding another layer to a layered cost map architecture like the one presented by Lu et al. (2014).

Although there are several works evaluating the utility of dynamics information in the literature, only a few focus on using maps of dynamics for motion planning (Mohanan and Salgoankar, 2018) or measure how the MoDs affect execution of planned motions (Palmieri et al., 2017; Swaminathan et al., 2018). In other words, it has been unclear to what extent MoDs are useful in global planning. Many works assume that it is possible to track and predict the motion of dynamic entities in the environment (Kruse et al., 1997; Bennewitz et al., 2005; Chung and Huang, 2011). These works emphasize the use of live dynamics information in the evaluation, thereby taking the focus off MoDs. Measuring the utility of MoDs is however difficult due to three main factors: 1) the lack of objective metrics; 2) the difficulty involved in reproducing and repeating real-world experiments, together with the difficulty of finding enough participants and ensuring that privacy and safety regulations are met; 3) the low fidelity of current simulators in simulating human-robot interaction. Experiments in simulations can however be repeated easily and a benchmarking platform based on such a simulation would reduce the time and effort required. To this end, in this paper we focus on the application of MoDs for global motion planning, and, in particular, quantitatively measuring their impact on the robot’s performance while executing motions in simulation.

The contributions of this paper are as follows:

1. We propose *five* novel cost functions for sampling-based motion planners using different MoDs.
2. We propose *two* novel objective metrics to evaluate the utility of MoDs to motion planning.
3. We develop a simulation framework for benchmarking the utility of MoDs to global motion planning and to facilitate reproducibility and development of further evaluations.
4. We compare *four* different MoDs based on their utility to global motion planning using the novel cost functions.

The paper is structured as follows. In Section 2, we provide a brief overview of MoD representations and summarize related work on motion planning using MoDs. The selected MoDs are representative to their specific classes thus constitute general enough evaluation. In Section 3, we recall sampling-based motion planning since it is an effective method for planning motions of generic wheeled robots in cluttered environments, while specifically focusing on the Rapidly Exploring Random Trees (RRT*) algorithm by Karaman and Frazzoli (2011). Although the proposed framework is general to motion planning algorithms, we use RRT* as a backbone algorithm since it is anytime optimal. Please note that we indeed aim to avoid bias in the evaluation due to the choice of a suboptimal planner (e.g., local minima) by ensuring that the planning duration is long enough. We detail in Section 4 how we combine traditional cost functions (path length and curvature) with cost functions for MoD-aware planning so as the proposed costs can be used with *any* motion planner (e.g., navigation functions, optimization-based solutions, or other sampling-based motion planners) using costs to compute paths. We provide a detailed description of the MoDs used in this work along with the proposed cost functions for the different MoDs in Section 5. We present our benchmarking method that assesses the performance of motion planning from the execution of planned paths in human-populated environments in Section 6. We describe the simulation framework we use to perform this benchmarking in Section 6.3. In Section 7, we detail the specifics of the experimental setup used in this work. We present a detailed discussion of the results in Section 8. Finally, in Section 9 we provide an outlook on future work and conclusions.

## 2 Related work

### 2.1 MoD types and examples

**FIGURE 2:**
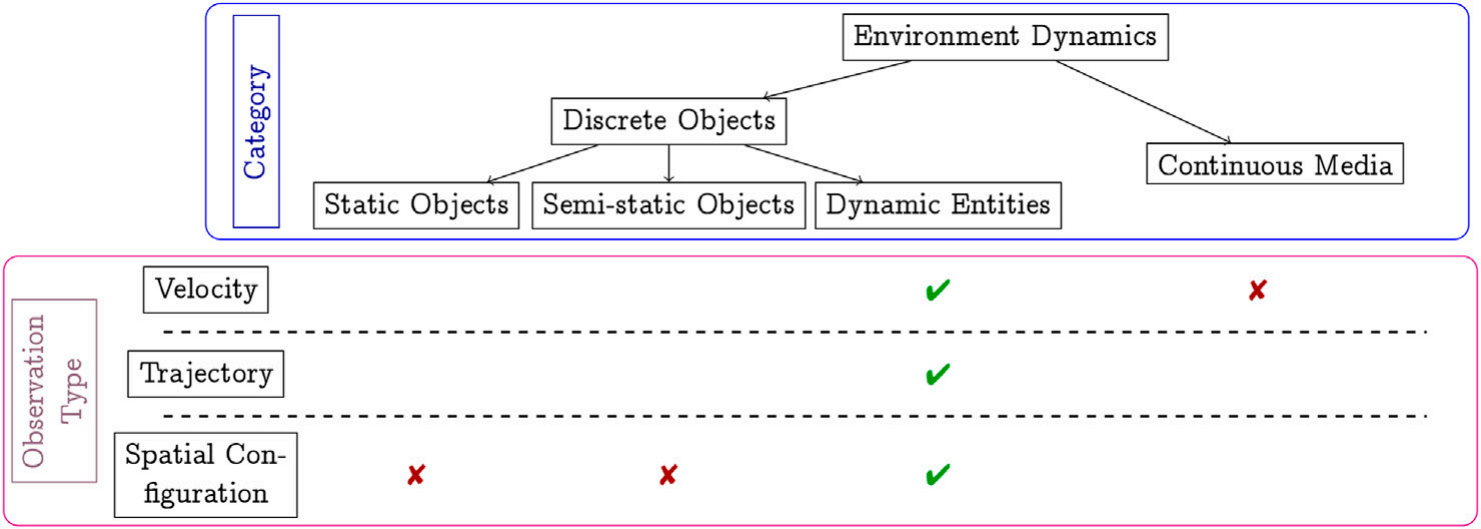
The figure shows the different categories of dynamics, and how they are observed (and mapped in consequence). The crosses and ticks show how different types of dynamics can be observed. The green ticks denote the areas of interest for this work.

MoDs can be classified into different groups based on *what class of dynamics* is being mapped. Figure 2 shows a categorization of dynamics based on the work by Kucner et al. (2020a). Several categories of dynamics are mentioned in Figure 2 and briefly discussed in the following. Static objects, such as walls and shelves, rarely change position over long periods of time. These are modeled using ordinary geometric maps [such as occupancy grids (Thrun, 2002), Octomaps (Hornung et al., 2013)] and are not a subject of MoDs. Semi-static objects, such as boxes, barricades, chairs, may change position with a relatively low frequency and as a consequence of specific events. Occupancy grid representations are common also when mapping semi-static objects. For example, the work in (Krajník et al., 2016) exploits the occupancy grid in combination with a the temporal model called FreMEn (Krajník et al., 2017), in order to model the state changes of the semi-static cells as a function of time. Semi-static objects are typically observed as changes in spatial configuration.

The defining property of dynamic entities, such as pedestrians, vehicles, or animals, is that they move purposefully and driven by the agents’ intentions. Dynamic entities may be observed by their velocity [Velocity fields, CLiFF-map Kucner et al. (2017)], trajectory (Kruse et al., 1997; Bennewitz et al., 2002; Ellis et al., 2009) or through spatial configuration changes.

MoD information of continuous media and dynamic entities is used in a qualitatively different manner when planning the motions of a robot. With continuous media (e.g., wind or water), the mapped dynamics describe the medium in which the robot operates. The planner may use the MoD information to minimize energy consumption and improve task efficiency: the robot may indeed slow down due to opposing water or air currents but may never get stuck.

With dynamic entities (e.g., pedestrians), the main goal of the planner is not only avoiding collisions, but also preserving task efficiency (e.g., by preventing the robot from getting stuck). This problem, known as the *Freezing Robot Problem* (Trautman and Krause, 2010), is a well-known problem in the robotics community. Although unable to provide formal guarantees, embedding MoD-information into planning should intuitively reduce the time robots need to navigate human-populated environments (as shown by the experiments in Section 8).

In this paper, we focus primarily on motion planning using MoDs that map dynamic entities such as pedestrians^1^. As seen in Figure 2, these are typically observed as velocities, trajectories or spatial configuration changes. We detail the common MoDs used to map pedestrians in Section 2.2.

### 2.2 Motion planning over MoDs of dynamic entities

Prior work involving MoDs of dynamic entities can be classified broadly into two groups: trajectory models (Section 2.2.1) and vector fields (Section 2.2.2). Trajectory models represent the motion patterns of the dynamic entities as a trajectory, whereas vector fields represent the velocity and/or direction of the entities at a certain location in space.

#### 2.2.1 Motion planning over trajectory models

Kruse et al. (1997) aim to address “statistical motion planning that respects typical obstacle behaviour in order to improve pre-planning in dynamic environments”. The dynamic obstacles are modelled as stochastic trajectories and a Poisson process is used to assign occurrence rates to each of them. The stochastic trajectories and the path of the robot are used to determine the probability of collision, which is used in motion planning. The work is further extended in Kruse and Wahl (1998): the workspace is represented as a 2D grid-map where each cell contains a set of eight transition probabilities related to adjacent cells. They evaluate the utility of MoDs to global motion planning and while assessing motion plans objectively, they estimate the time to reach the goal. In contrast, our work focuses on calculating the actual time wasted by executing motions on a robot while replaying recorded pedestrian data.

Bennewitz et al. (2005) model typical patterns of human motion using Gaussian Mixture Models of trajectories (GMMT). Each motion pattern is represented by a set of Gaussian distributions. These are estimated from observed trajectories using the Expectation-Maximization algorithm. From the motion patterns, Hidden Markov Models (HMM) are derived. At the time of planning, HMMs are used to forecast the trajectories of the observed people by using live dynamics instead of motions encoded in the GMM map. In this paper we define a cost function for the GMMT-map and compare it with other MoDs.

Similarly, Fulgenzi et al. (2008) also utilize trajectory-based MoDs in motion planning. They use Gaussian Processes to model the trajectories of dynamic entities and then use them to predict the future motions given the observations. These predictions are then used to compute a path that is most probable to avoid collisions. The work by Fulgenzi et al. (2009) uses a similar idea but employs HMMs to represent the moving entities. Aoude et al. (2013) discuss the use of Gaussian Process models to represent the motion patterns of objects. The future positions of detected objects are predicted and then used in a modified RRT algorithm called chance-constrained RRT (Luders et al., 2010). These works also employ the use of live dynamics information. In contrast, we focus on the use of MoDs in motion planning in this paper.

#### 2.2.2 Motion planning over velocity models

Ko et al. (2014) propose an extension to the Rapidly-exploring Random Trees (RRT) algorithm called the VFRRT (Vector Field RRT). The idea of an *Upstream Criterion* is introduced. The Upstrem Criterion is a cost function defined such that motions along the flow have lower cost than motions against the flow. This used to bias the search so that the probability of finding flow-conforming paths is maximized. In essence, VFRRT finds paths with low Upstream Criterion, that are paths that require low control effort. Palmieri et al. (2017) propose the CLiFF-RRT* algorithm using a modified Upstream Criterion. The cost function comprises two parts: a cost due to path length [a distance metric is employed as done by LaValle and Kuffner (2001)] and a cost due to compliance with the speed and direction encoded in the MoD (CLiFF-map). The CLiFF-RRT* cost function does not utilize the covariances available in the CLiFF-map. On the other hand, the DTC-RRT* algorithm by Swaminathan et al. (2018) proposes a more general cost function using all components of the CLiFF-map to compute the MoD cost. The approach employs the Mahalanobis distance to cost trajectories based on how much the velocity of the robot complies with the underlying MoD. Both these works evaluate the usefulness of MoD-aware motion planning in terms of quality of the resulting trajectories. Results in simple scenarios demonstrate the effectiveness of embedding CLiFF-maps into global motion planning. However, both works rely on simulated data to generate the MoD (CLiFF-map). Conversely, in this work, we use real pedestrian data to generate the MoD. Also, we here propose metrics based on the execution of the motion plans to objectively gauge the utility of MoDs to global planning.

Motion planning over MoDs of fluid media is considerably different from motion planning over MoDs that models dynamic entities. However, MoDs of fluid media can be used to capture flow patterns of discrete entities in an environment. Thus, these approaches provide valuable ideas for designing motion planners of robots accounting for e.g., flow of pedestrians in a road.

### 2.3 Comparisons of MoDs-informed motion planners

In a previous collaborative effort, Vintr et al. (2020) propose a method for the evaluation of MoD-aware motion planning. Motion planning is performed on a finite grid using graph-search. Then, recorded data is played back alongside a robot executing its motions. The number of interactions between the robot and pedestrians are counted and used as a part of the evaluation. Stuede and Schappler (2022) also compute the number of encounters between the robot and humans similar to the work by Vintr et al. (2020).

In contrast to both these works, we use sampling-based motion planners that are more popular in real world applications with non-holonomic robots. Notice that the pedestrians do not respond to the robot in the framework described by Vintr et al. (2020). In this respect, we utilize a more extensive simulation framework for the simulation where the robot and pedestrians alike, pause execution at intersections in order to let the others pass. In addition to that, even though we only use one robot in this paper, the simulation framework we propose can accommodate more than one robot.

Chen et al. (2018) present a framework for human-aware robot navigation. In their evaluation framework, they describe two possibilities. In one, the humans only react to other humans and not the robot. This is similar to the framework by Vintr et al. (2020) although in the latter’s work, recorded pedestrian data is replayed. In the second, the visible-robot setting, both humans and robots react to each other. However, they do not consider robots or humans simply stopping to let the other pass by. In this work, we deliberately utilize the aforementioned “stopping to let the other pass by” because we want to evaluate the original paths and, therefore, avoid modifying them.

## 3 Sampling-based motion planning

Planning the motion of wheeled vehicles operating in real applications requires dealing with differential constraints such as bounds on velocity and acceleration and non-holonomic constraints. Approaches that resort to constrained optimization to find a feasible path among two locations are generally not as practical as sampling-based motion planners (Yang et al., 2019; Heiden et al., 2021). Indeed, the presence of obstacles in the environment may introduce non-convex constraints in the optimization problem which further affect its complexity (Schoels et al., 2020; Zhang et al., 2020). Although sampling-based motion planners do not guarantee finding the optimal solution to a motion planning problem in finite time, they produce reasonable solutions quickly also in cluttered environments and can deal with kinematic and dynamic constraints. This makes these planners well-suited (and hence widely used) in practice to plan paths of robots moving in real environments and subject to differential constraints (such as of wheeled robots). In this paper, we focus on using sampling-based motion planners for this reason.

In Sections 4 and 5, we describe how to incorporate MoD information in traditional sampling-based motion planning algorithms using an MoD cost function in addition to traditional cost metrics. Notably, the cost functions described in this paper can be used with any type of motion planner. For clarity, we briefly recall the RRT* algorithm in Section 3. Then, in Section 4, we present our approach to incorporating MoD information in motion planning.

We consider a robot whose kinematics are described by the differential equations $\dot{x}\left(t\right)=\text{f}\left(x\left(t\right),u\left(t\right)\right)$ where **x**(*t*) is the robot configuration and **u**(*t*) are the robot controls at time *t*. For instance, the kinematic model of a non-holonomic wheeled vehicle operating in a flat 2D environment can be described by the vectors **x** = (*x*, *y*, *θ*) and **u** = (*υ*, *ω*), where **r** = (*x*, *y*) is the robot position (relative to a fixed world frame), *θ* is the heading, *υ* is the linear velocity of the robot, and *ω* is its angular velocity. Let $X\subset R^{n}$ the configuration space, $U\in R^{m}$ be the control space of the robot. Also, let $X_{\text{obs}}$ be the obstacle space and $X_{\text{free}}\subseteq X\backslash X_{\text{obs}}$ be the free space. Given a starting configuration **x**_start_, and a goal region $X_{\text{goal}}\subset X_{\text{free}}$ , *motion planning* is the problem of computing a trajectory ***β*** = (***β***_1_, ***β***_2_, … , ***β***_*f*_) = ((**x**_1_, **u**_1_), (**x**_2_, **u**_2_)…, (**x**_*f*_, **u**_*f*_)) such that **x**_1_ = **x**_start_, $x_{f}=x_{\text{goal}}\in X_{\text{goal}}$ , $x_{i}\in X_{\text{free}}\;\forall i\in \left\{1,2,\ldots ,f\right\}$ , and ***β*** obeys to the robot kinematics and satisfies kinematic and dynamic constraints. Let **p** = (**x**_1_, **x**_2_, … , **x**_*f*_) be the related path.

The RRT* algorithm by Karaman and Frazzoli (2011) is a probabilistically complete, asymptotically optimal, single-query sampling and searching algorithm. Given a starting configuration **x**_start_, the free space $X_{\text{free}}$ , and a goal region $X_{\text{goal}}\subset X_{\text{free}}$ , RRT* creates a tree *τ* in $X_{\text{free}}$ (rooted in **x**_start_) by incrementally sampling states in $X_{\text{free}}$ . Nodes in the tree correspond to states and edges correspond to kinematically feasible motions (paths or trajectories) between states. The motion planning problem is hence translated into finding the min-cost path in the tree *τ* from **x**_start_ to $X_{\text{goal}}$ . The RRT* algorithm is shown in Algorithm 1.

The treeis first initialized with the start state (line 1). A new state **x**_rand_ is randomly sampled *via* a sampling mechanism defined by the Sampling() function (line 5). Then, Nearest Search (*τ*, **x**_rand_) is used to find the node **x**_near_ in the tree that is closest to **x**_rand_ (line 6). A local planning method defined by Extend (**x**_near_, **x**_rand_) is used to find a feasible motion ***δ*** connecting **x**_near_ to a state **x**_new_ in the proximity of **x**_rand_. This state corresponds to **x**_rand_ whenever a perfect connection is possible, or, to a new state conversely. The cost of a path to **x**_new_ from **x**_start_ is then initialized (line 13). Then the tree is rewired to ensure all nodes are reached with minimum-cost paths (line 14). Please refer to the work by Karaman and Frazzoli (2011) for a more detailed explanation.

## 4 Cost functions for MoD-aware motion planning

### 4.1 Cost function components

In this section, we focus on the cost function cost(***δ***) in Algorithm 1. Similar to the work by LaValle and Kuffner (2001), we design this function so as the planner will aim at minimizing the length and maximizing the smoothness of the returned motion.

Algorithm 1 RRT* algorithm.

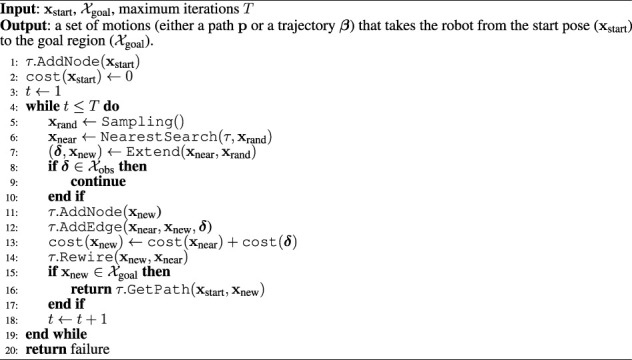

Let**r**_*i*_ and **q**_*i*_ be the position and the quaternion associated with the trajectory point ***β***_*i*_, respectively. For each trajectory point ***β***_*i*_, we model the effort of moving from ***β***_*i*−1_ to ***β***_*i*_ as the sum of the Euclidean cost *c*_*d*_(***β***_*i*_) = ‖**r**_*i*_ − **r**_*i*−1_‖ and the quaternion cost $c_{q}\left(\beta_{i}\right)=1-{\left(q_{i}^{\text{T}}q_{i-1}\right)}^{2}$ . The Euclidean distance cost *c*_*d*_(***β***) and quaternion distance cost *c*_*q*_(***β***) of a trajectory ***β*** are then computed as $c_{d}\left(\beta \right)=\sum_{i=2}^{a}c_{d}\left(\beta_{i}\right)$ and $c_{q}\left(\beta \right)=\sum_{i=2}^{a}c_{q}\left(\beta_{i}\right)$ . Thus, *c*_*d*_(***β***) ≥ 0 and *c*_*q*_(***β***) ≥ 0 by construction.

To incorporate MoD information, we introduce an additional MoD-dependent cost *c*_*c*_ into the total cost *c*_tot_ returned by the function cost(***δ***). In addition to our previous CLiFF-RRT* and DTC-RRT* cost functions (Palmieri et al., 2017; Swaminathan et al., 2018), in this paper we propose a new definition of the cost function *c*_*c*_ suitable also for STeF-maps, GMMT-maps and Intensity-maps, as detailed in Section 5. All MoD costs are designed to have lower values when a robot trajectory adheres to the flow modeled *via* the MoD and higher values otherwise. We can then define the total cost of a trajectory as the weighted sum of the different components:

$$c_{\text{tot}}\left(\beta \right)=w_{d}c_{d}\left(\beta \right)+w_{q}c_{q}\left(\beta \right)+w_{c}c_{c}\left(\beta \right),$$ (1)

where *w*_*d*_, *w*_*q*_ and *w*_*c*_ are weights for the costs due to the Euclidean distance, the quaternion distance and MoD, respectively. If *w*_*d*_ is set to zero, the distance metric is ignored and the resulting trajectories are strictly flow-conforming (or flow-flouting, depending on how *c*_*c*_ is formulated). If *w*_*c*_ is set to zero, the planner behaves as a regular or MoD-unaware RRT*.

### 4.2 Selection of weights for cost function components

In this section, we discuss how the weights for each MoD can be chosen. Although it is impossible to equate the costs due to each MoD individually, the weights can be chosen so that the total cost (distance cost and MoD-cost) is equivalent. An optimizing motion planner (such as RRT*) would find an initial solution and then try to improve it. We would like to show here that a planner would select a motion plan for improvement that is at most *γ* times longer than the initial path found, for the chosen weights.

Suppose the initial motion plan is ***β*** ^(*i*)^. For simplicity, yet without loss of generality, let us assume that *w*_*q*_ = *w*_*d*_ = 1. The cost of such a plan is *c*_tot_(***β*** ^(*i*)^) = *c*_*d*_(***β*** ^(*i*)^) + *c*_*q*_(***β*** ^(*i*)^) + *w*_*c*_ *c*_*c*_(***β*** ^(*i*)^)

Now consider a candidate solution ***β*** ^(*f*)^. We know that ***β*** ^(*f*)^ improves the initial motion plan ***β*** ^(*i*)^ only if *c*_tot_(***β*** ^(*f*)^) < *c*_tot_(***β*** ^(*i*)^). This condition places an upper limit on the Euclidean cost (and hence on the length considering our definition of *c*_*d*_) of the candidate solution ***β*** ^(*f*)^ since *c*_*d*_ (***β*** ^(*f*)^) ≤ *c*_tot_(***β*** ^(*f*)^) < *c*_tot_(***β*** ^(*i*)^). In other words, if the planner returns the trajectory ***β*** ^(*f*)^, then its length is limited by *c*_tot_(***β*** ^(*i*)^).

Suppose the worst case MoD-cost per meter is *μ*, that is, *c*_*c*_(***β*** ^(*i*)^) ≤ *μc*_*d*_ (***β*** ^(*i*)^). Suppose that *c*_*q*_ ≈ 0, without loss of generality, we can then write: *c*_tot_(***β*** ^(*i*)^) ≤ (1 + *w*_*c*_ *μ*)*c*_*d*_(***β*** ^(*i*)^). Thus, the bounding constraint stated before (i.e., *c*_*d*_ (***β*** ^(*f*)^) < *c*_tot_(***β*** ^(*i*)^)) can be formulated as

$$c_{d}\left(\beta^{\left(f\right)}\right)<\left(1+w_{c}\mu \right)c_{d}\left(\beta^{\left(i\right)}\right)\text{, that is }c_{d}\left(\beta^{\left(f\right)}\right)<\gamma c_{d}\left(\beta^{\left(i\right)}\right),$$

which validates our claim. Since the cost functions we use all have different *μ*, i.e., different worst-case MoD-cost per metre, we use the same *γ* to calculate weights (*w*_*c*_) for each of the cost functions we use.

## 5 Representations and cost functions

In this paper, we use one MoD from each category shown in Figure 2: one trajectory map (GMMT-map), one velocity map (CLiFF-map), one based on spatial configuration (Intensity-map) and one based on the likelihood of motion directions on a grid-based map (STeF-map). STeF-maps can be thought of as modeling spatial configuration changes with respect to direction. Additionally, STeF-maps model the periodicities associated to such likelihoods. Although we choose one MoD from each category, any other existing representation can be used in its place as long as it represents available information about entity dynamics.

### 5.1 CLiFF-map

#### 5.1.1 CLiFF-map description

The Circular-Linear Flow Field (CLiFF) map, proposed by Kucner et al. (2017), allows us to model velocities probabilistically. The CLiFF-map model associates a probability distribution over velocity (direction and speed) to each location in the map. These probability distributions are Gaussian mixture models. The mixture model is suitable for describing the motion of dynamic entities. For example, at road intersections, velocities of cars are typically along more than one principal direction. A mixture model also allows us to capture such variations in motion. The locations in a CLiFF-map could be regularly-spaced locations on a grid or arbitrary locations, where velocity information was recorded. Additionally, the CLiFF-map can be built from data that is incomplete or spatially sparse. This makes it particularly suitable for mapping of dynamic media such as wind and water since measurements can only be obtained sparsely in a realistic scenario. However, the datasets described in this paper are recorded with complete observability.

CLiFF-map’s probability distributions capture both the uncertainty in sensing and inherent variability of the velocities. The random variable, velocity, is described in polar coordinates as a 2D vector of the heading, *θ* and speed, *ρ*, as follows: $V={\left[\begin{array}{c} \theta \;\;\rho \end{array}\right]}^{\text{T}}$ , where *θ* ∈ [0, 2*π*) and *ρ* ∈ [0, *∞*). This allows each component to be interpreted independently as opposed to a Cartesian representation of the velocity.

The Probability Density Function (PDF) of a mixture model can be written as

$$p\left(V\right)=\overset{J}{\underset{j=1}{\sum }}\pi_{j}f_{j}\left(V\right),$$ (2)

where *f*_*j*_ represents the *j*-th base distribution in the mixture. The mixing factor *π*_*j*_ is the fraction of observations that belong to the *j*-th distribution in the mixture.

Since the random variable, velocity, is described in polar form, the base distribution in the mixture is represented by a semi-wrapped Normal distribution (SWND). An SWND is a bi-variate Gaussian distribution of the velocity, where one of the dimensions (the heading in CLiFF-map) is wrapped around the unit circle. This can be written as:

$$f_{j}\left(V\right)=N_{\Sigma_{j},\mu_{j}}^{SW}\left(V\right)=\underset{k\in Z}{\sum }N_{\Sigma_{j},\mu_{j}}\left(\left[\begin{array}{c} \theta \\ \rho \end{array}\right]+2\pi \left[\begin{array}{c} k\\ 0 \end{array}\right]\right),$$ (3)

where ***μ***_*j*_, **Σ**_*j*_ and *k* are the mean, covariance and the winding number of the *j*-th distribution over **V**, respectively.

In addition to the aforementioned probability distributions, CLiFF-map also models information related to how long a location was observed. The duration for which a location was observed is modelled as a proportion of the total observation time. Let us consider an example to elucidate the utility of this quantity. Consider an environment where autonomous robots are to be deployed. The constraint is that we desire little or no modification of the existing environment. Therefore, all sensors are placed on the autonomous robot. We wish to sense dynamic entities and collect data pertaining to their velocities. This means that each location will be observed for a different duration of time. Additionally, during the time that each location is observed, motion might be observed only for a portion of that.

The *motion ratio*, $q_{s}=\frac{T_{s}^{m}}{T_{s}^{o}}$ , where $T_{s}^{m}$ is the time during which motion was observed at the location and $T_{s}^{o}$ is the time during which the location was observed. A motion ratio of one indicates that whenever this location was observed, motion was also observed. A zero motion ratio indicates that motion was never observed in that location. The *observation ratio*, *p*_*s*_, is the ratio of $T_{s}^{o}$ and the total observation duration for all locations (*T* ^*t*^): $p_{s}=\frac{T_{s}^{o}}{T^{t}}$ . An observation ratio of one indicates that the location was observed for the entire duration of observation, whereas a value less than one indicates partial observation, and a value of zero indicates no observation.

In summary, a CLiFF-map in 2D space can be denoted as

$$\Xi =\left\{\left(\xi_{s},p_{s},q_{s},l_{s}\right)|s\in Z^{+}\wedge l_{s}\in R^{2}\right\}$$ (4)

where ***ξ***_*s*_ is the set of parameters describing the distribution with *J*_*s*_ mixtures at location **l**_*s*_, that is, $\left(\left(\pi_{1},\Sigma_{1},\mu_{1}\right),\left(\pi_{2},\Sigma_{2},\mu_{2}\right),\ldots ,\left(\pi_{J_{s}},\Sigma_{J_{s}},\mu_{J_{s}}\right)\right)$ .

#### 5.1.2 Down-the-cLiFF cost

In this section we describe the Down-The-CLiFF cost function first introduced in our previous work (Swaminathan et al., 2018). The DTC cost function aims to penalize trajectories where the robot’s velocity deviates from the CLiFF-map probability distribution. The Mahalanobis distance is a primary component of the cost function since it measures how close an instance is from the underlying distribution. The cost described below is a normalized version of the cost described in our previous work. As we already discussed in Section 4.2, normalization is done to facilitate easy and meaningful selection of cost function weights.

Suppose $\upsilon_{i}^{\text{pol}}$ is a velocity vector in polar coordinates, i.e., it is composed of the heading and speed associated with the trajectory point. Suppose there are *J*_*i*_ SWNDs at the CLiFF-map location corresponding to the position of the trajectory point ***β***_*i*_. The cost of a trajectory point is the weighted sum of Mahalanobis distances due to all SWNDs:

$$c_{c}\left(\beta_{i}\right)=D_{i}=\overset{J_{i}}{\underset{j=1}{\sum }}\left(\pi_{j}\sqrt{{\left(\upsilon_{i}^{\text{pol}}-\mu_{j}\right)}^{T}\Sigma_{j}^{-1}\left(\upsilon_{i}^{\text{pol}}-\mu_{j}\right)}\right).$$ (5)

We define the Down-The-CLiFF (DTC) cost for a trajectory as a weighted sum of all trajectory points:

$$c_{c}\left(\beta \right)=\overset{a}{\underset{i=1}{\sum }}\left(D_{i}\right).$$ (6)

Another version of this cost utilizes the motion ratio *q*_*i*_ available in the CLiFF-map:

$$c_{c}\left(\beta \right)=\overset{a}{\underset{i=1}{\sum }}\left(q_{i}D_{i}\right).$$ (7)

*q* weighting in Eq. 7 would lead to lower costs for locations with less motion. Hence, a planner using this cost function will generate trajectories along regions with less motion (*congestion-avoiding* due to *q*), while simultaneously trying to match the speed and orientation of the underlying flow (*flow-conforming* due to Mahalanobis distance), where more motion was observed.

We use both (Eqs 6 and 7) cost functions in this paper to better distinguish the utility of different types of MoD information available. We limit the maximum value of Mahalanobis distance to 10, which enables us to limit the maximum cost at a trajectory point.

#### 5.1.3 Extended upstream criterion

Another alternative for the MoD cost *c*_*c*_ in Eq. 1 is defined by Palmieri et al. (2017). The *Extended Upstream Criterion* (EUC) is an extension to the upstream criterion proposed by Ko et al. (2014). Analogous to Eq. 5, the EUC associated with a trajectory point ***β***_*i*_ can be written as:

$$U_{i}=\overset{J_{i}}{\underset{j=1}{\sum }}\left(\|{\vec{\mu }}_{j}\|-\left({\vec{\mu }}_{j}\cdot {\hat{n}}_{i}\right)\right),$$ (8)

where ${\vec{\mu }}_{j}$ is the CLiFF-map mean (in Cartesian coordinates) at the location corresponding to trajectory point ***β***_*i*_, and ${\hat{n}}_{i}=\frac{\upsilon_{i}}{\left\|\upsilon_{i}\right\|}$ is the unit tangent vector along the velocity direction at the trajectory point ***β***_*i*_. For consistency with Eq. 5, we also modify the expression for EUC above to include the mixing factor *π*_*j*_ as follows. Again, this enables us to easily limit the maximum value of cost (EUC) at a trajectory point.

$$c_{c}\left(\beta_{i}\right)=U_{i}=\overset{J_{i}}{\underset{j=1}{\sum }}\left(\pi_{j}\cdot \left(1-\cos\alpha \right)\right)$$ (9)

where *α* is the angle between the vectors ${\vec{\mu }}_{j}$ and ${\hat{n}}_{i}$ .

We use the motion ratio *q* to construct an alternative cost function similar to Eq. 7.

$$c_{c}\left(\beta_{i}\right)=U_{i}=q_{i}\overset{J_{i}}{\underset{j=1}{\sum }}\left(\pi_{j}\cdot \left(1-\cos\alpha \right)\right)$$ (10)

### 5.2 STeF-map

#### 5.2.1 STeF-map description

STeF-Map (Molina et al., 2018), which stands for Spatio-Temporal Flow Map, is a representation that models the likelihood of motion directions on a grid-based map by a set of harmonic functions, which capture long-term changes of crowd movements over time.

##### 5.2.1.1 Spatial representation

The underlying geometric space is represented by a grid, where each cell contains *k* temporal models, corresponding to *k* discretized orientations of people motion through the given cell over time. Since the total number of temporal models, which are of a fixed size, is *k* × *n* where *n* is the total number of cells, the spatio-temporal model does not grow over time regardless of the duration of data collection. This allows the model to make probabilistic predictions of the likely flow of people in a certain direction for a given cell at any instant of time.

##### 5.2.1.2 Temporal framework—FreMEn

The temporal models, which can capture patterns of people movement, are based on the FreMEn framework (Krajník et al., 2017). FreMEn is a mathematical tool based on the Fourier Transform, which considers the probability of a given state as a function of time and represents it by a combination of harmonic components. The model not only allows representation of environment dynamics over arbitrary timescales with constant memory requirements, but also prediction of future environment states based on the patterns learned. The idea is to treat a measured state as a signal, decompose it by means of the Fourier Transform, and obtain a frequency spectrum with the corresponding amplitudes, frequencies and phase shifts. Then, transferring the most prominent spectral components to the time domain provides an analytic expression representing the likelihood of that state at a given time in the past or future. Assuming that the directions of people movement are affected by patterns that might be periodic, STeF-map applies the FreMEn concept to discretized directions of people movement through a particular cell.

##### 5.2.1.3 Building the model

The STeF-map model assumes that it is provided with people detection data, containing person position, orientation and timestamp of the detection (*x*, *y*, *α*, *t*). At the beginning of the model construction, each cell is associated with *k* bins, corresponding to the discretized orientation of people motion, each with an associated temporal model. When building the model, the *x*, *y* positions are discretized and assigned to the corresponding cell and the orientation *α* is assigned to one of the *k* bins, whose value is incremented by 1. In other words, the number of people detections occurring in each orientation bin of each cell is counted. After a predefined interval of time, the bins are normalized, and the normalized values are used to update the spectra of the temporal models by the scheme described by Molina et al. (2018) in their work. Then, the bin values are reset to 0 and the counting again is started again. Notice that when building the model the total number of detections in each cell is not used, only the relative number of occurrences among all *k* orientations.

##### 5.2.1.4 Making predictions

To predict the behaviour of human movement through a cell at a future time *t*, the probability for each discretized orientation *θ*, ( $\theta =i\frac{2\pi }{k}$ and *i* ∈ {0 … *k* − 1}), associated to that cell is calculated as

$$p_{\theta }\left(t\right)=p_{0}+\overset{m}{\underset{j=1}{\sum }}p_{j}\cos\left(\omega_{j}t+\varphi_{j}\right),$$ (11)

where *p*_0_ is the stationary probability, *m* is the number of the most prominent spectral components, and *p*_*j*_, *ω*_*j*_ and *φ*_*j*_ are their amplitudes, periods and phases.

The spectral components *ω*_*j*_ are drawn from a set of *ω*_*s*_ that covers periodicities ranging from hours to 1 week with the following distribution:

$$\omega_{s}=\frac{7\cdot 24\cdot 3600}{1+s},\;s\in 0,1,2,3,4,\ldots ,15.$$ (12)

The choice of *m* determines how many periodic processes are considered for prediction. Setting *m* too low could mean omitting other less prominent environment processes, while setting it too high might decrease the generalization capabilities of the model.

#### 5.2.2 STeF-map cost function

Analogous to Eq. 9, we define the Extended Upstream Criterion for STeF map as follows:

$$c_{c}\left(\beta_{i}\right)=U_{i}=\overset{K}{\underset{k=1}{\sum }}\left\{p_{k}\left(1-\cos\left(\alpha -\left(k-1\right)\frac{\pi }{4}\right)\right)\right\}$$ (13)

where *α* is the heading angle associated with the trajectory point ***β***_*i*_, and *p*_*k*_ is the predicted probability provided by the STeF-Map model of finding people moving for each of the *k* orientations in the location associated with the destination node. For all the experiments, the value of *k* was set to 8.

### 5.3 GMMT-map

#### 5.3.1 GMMT-map description

In this section, we present the probabilistic modelling technique proposed by Bennewitz et al. (2002), which we refer to as GMMT-map (Gaussian Mixture Model Trajectory map). The GMMT-map aims to model trajectories of persons using the following intuition: when humans move around in an environment our motion is not random. Instead, we tend to exhibit typical patterns related to our activity or destination. Thus, the trajectories are modelled as motion patterns described by probability distributions.

The input data used to build the GMMT-map is a collection of trajectories, where each trajectory is a sequence of 2D positions. When the model is built, the data is clustered into *M* different motion patterns. Each motion pattern is represented by a sequence of K Gaussian distributions.

Suppose ***μ***_*mk*_ is the mean position of the *k*-th point along the *m*-th motion pattern and ***σ*** is the standard deviation along both *x* and *y* directions. The GMMT-map can be written as a mixture model with *M* components as

$$p\left(\tau \right)=\sum_{m}\pi_{m}\overset{K}{\underset{k=1}{\prod }}N\left(\mu_{mk},\sigma \right),$$ (14)

where ***τ*** is the random variable describing the trajectory and *π*_*m*_ is the mixing factor of the *m*th motion pattern or cluster. *π*_*m*_ signifies the proportion of total trajectories that belong to motion pattern *m*. The base distribution in the mixture model is a product of Gaussians. It is also the probability distribution describing the *m*th motion pattern and can be written as

$$p\left(z\right)=\overset{K}{\underset{k=1}{\prod }}N\left(z|\mu_{mk},\sigma \right).$$ (15)

A modified EM algorithm is used to build the GMMT-map where the means are iteratively improved. The input to the EM algorithm is a set of trajectories, each consisting of the positions and timestamps of the observed pedestrian or entity. In the original work by Bennewitz et al., the mixing factors are not computed. Thus, the expression for recursively computing the responsibilities implicitly assumes that the mixing factors for all mixtures are equal, in the original paper. In contrast, we compute the mixing factors and use them to compute responsibilities.

#### 5.3.2 GMMT-map cost function

Analogous to previous sections, we find a suitable cost function that uses the GMMT-map to compute the MoD cost (*c*_*c*_ from Eq. 1). We utilize the EUC modified specifically for the GMMT-map as shown in Eq. 16. The cost is zero if the trajectory point lies farther away than one standard deviation from a Gaussian.

$$U_{i}=\overset{m}{\underset{m=1}{\sum }}\pi_{m}b_{mt}\;\left(1-\sigma^{-1}\|\mu_{mt}-r_{i}\|\right)\;\left(1-\cos\alpha_{mt}\right),$$ (16)

where

$$b_{mk}=\left\{\begin{array}{cc} 1:\; & \|\mu_{mk}-r_{i}\|<\sigma ,\\ 0:\; & \text{otherwise}. \end{array}\right.$$

In Eq. 16, ***μ***_*mt*_ is the mean of the *m*-th motion pattern that is closest to **r**_*i*_; **r**_*i*_ is a vector describing the position at ***β***_*i*_; *α*_*mt*_ is the angle between the heading at ***β***_*i*_ and the direction of the *m*-th motion pattern at its *t*-th Gaussian (the Gaussian closest to **r**_*i*_). The direction of *m*-th motion pattern at its *k*-th point is an implicit quantity in the GMMT-map since the *K* Gaussians are ordered by the index *k* in each of the *M* motion patterns. Once the closest Gaussian is identified, the direction is used to compute the cosine distance. The sum of *U*_*i*_ over all trajectory points gives us the cost due to GMMT-map for the entire trajectory similar to Eq. 6, and is omitted in this section.

### 5.4 Intensity-map

#### 5.4.1 Intensity-map description

Intensity map is the simplest MoD in our analysis. Similar to CLiFF-map and STeF-map, we use a regularly spaced grid. At each cell *i* in the grid the intensity is defined as

$$Q_{i}=\frac{n_{i}}{\max\left(n_{0},n_{1},n_{2},\ldots ,n_{N}\right)},$$ (17)

where *n*_*i*_ is the number of observations at cell *i* and *N* is the total number of cells in the grid. Note that the normalization using the maximum value of observations is used to limit the maximum intensity value to 1.

#### 5.4.2 Intensity-map cost function

The MoD-cost (*c*_*c*_) due to intensity map is simply the value of intensity at the position corresponding to the trajectory point. That is, the Intensity cost *I*_*i*_ at trajectory point ***β***_*i*_ is defined as

$$I_{i}=Q\left(r_{i}\right)=Q_{j},$$ (18)

where **r**_*i*_ is the position associated with trajectory point ***β***_*i*_ as before, and *j* is the cell where point **r**_*i*_ falls in the grid.

## 6 Evaluation method

In the previous sections, we have detailed contributions 1 and 2 of this paper (see Section 1). We now detail our simulation framework for benchmarking the utility of different MoDs for global motion planning (contributions 3 and 4). The experiments are split into one preparatory phase (MoD-building) and two evaluation phases:

1. MoD-building: we use data to build the four different MoDs described in Section 5.
2. Planning: we use the representations built in the previous step in our loosely-coupled planning architecture to generate dynamics-aware paths between sets of predefined start and goal locations.
3. Execution: we use the multi-agent coordination framework (Section 6.3) to simulate the execution of the motion plans while replaying recorded pedestrian data and record the impact of human behaviour on the execution of the planned paths (see performance indices in Section 6.2).

The code supporting these steps is open-source. Instructions are available at https://ksatyaki.github.io. We explain the general details of the evaluation method: experiment setup and evaluation metrics. In the following section (Section 7) we describe the different parameters used in our experiments.

### 6.1 Planning phase metrics

During the planning phase we record the cost of solutions resulting from each of the motion planners. For each solution, we record the Euclidean distance cost (*c*_*d*_), quaternion distance cost (*c*_*q*_), and MoD cost (*c*_*c*_) (see Eq. 1). The main aim of these metrics is to motivate the need for the experiments based on execution of trajectories. In other words, although the metrics from the planning phase show some important characteristics of the planned motions, it is not enough to gauge the utility of MoDs to motion planning per se.

### 6.2 Execution phase metrics

Recall that the objective of this study is not only to gauge the utility of MoDs to motion planning, but also to compare the different MoDs based on their utility to global planning. We propose to do this by measuring the time wasted by the robot and pedestrians as the robot executes the planned motions alongside the pedestrians. We also expect to see what type of MoD information is more valuable with respect to reducing the time wasted. Similarly, we could compare the execution of motions from all the different planners.

Since we wish to measure the utility of execution of motions generated by the global planners, we limit local planning in order to measure the effect of global planning alone. That is, the local planner does “as little as possible” by pausing the execution of motions, thereby not modifying the original path. In other words, replanning is not considered because we want to guage the original motion plans and not the replanned motions. Thus, in our case the local planner behaves as follows: the robot should simply pause execution when a human is about to cross its path. The robot resumes execution along the path when the human has passed.

Notice that instead of the robot pausing to let the human pass-by, a human could pause to let the robot pass-by. In essence, three possibilities exist: 1) adamant human, 2) adamant robot and 3) cooperative agents. Adamant humans means that humans always gets precedence and the robot always waits. Adamant robot is the opposite behaviour, where robots are given precedence. In real scenarios, similar to human interactions, we might expect that whoever is closer to the intersection point would pass-by while the other one waits. That is, the agents engage in a cooperative manner. In our experiments we test only the cooperative strategy owing to the high number of experiments.

In summary, we measure two quantities in order to objectify the utility of MoDs to motion planning: *time wasted* and *success rate* of execution. We measure the time wasted by calculating the duration a robot or pedestrian waits for the other to pass-by or crossover as already described in the previous section.

$$t_{\text{waste}}=t_{\text{waste, robot}}+t_{\text{waste, pedestrian}}$$ (19)

When robots or humans get stuck waiting for eachother, replanning is necessary in order to continue execution. In our simulator based on the coordination-framework, a deadlock has occurred when replanning is necessary (discussed further in Section 6.3). Therefore, success rate of execution is the percentage of executions that did not result in a deadlock, i.e., where replanning was not necessary.

In order to simulate and to perform our experiments we make use of a multi-agent coordination framework, which we discuss next.

### 6.3 Multi-agent coordination framework

**FIGURE 3:**
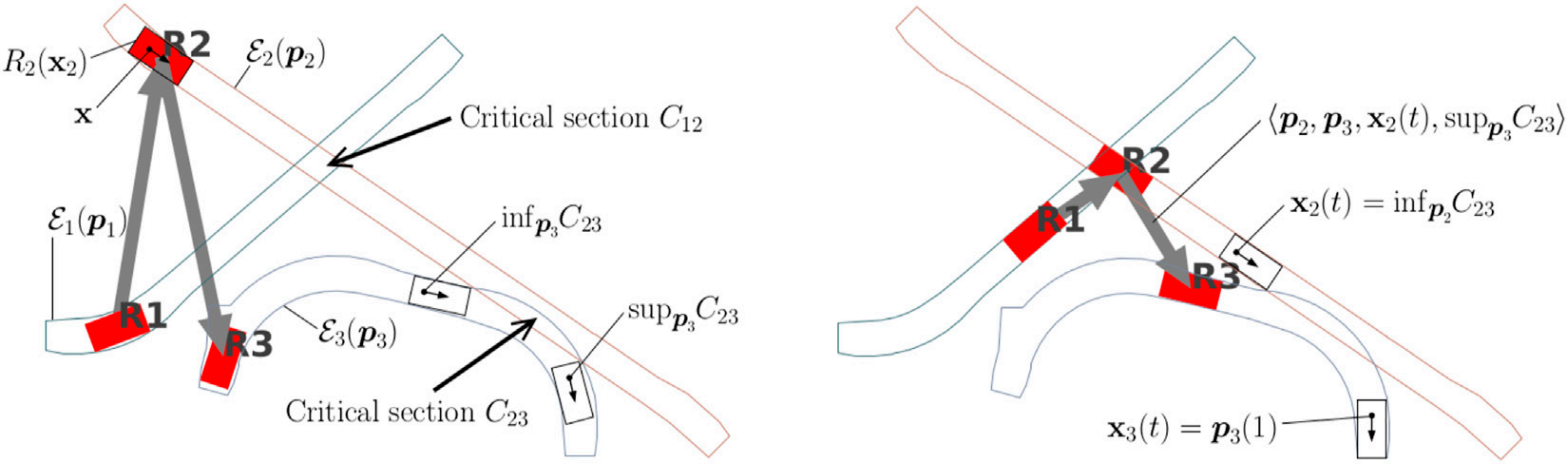
Three robots navigating along paths p_1_, p_2_, and p_3_. Spatial envelopes and critical sections are shown on the left; gray arrows indicate precedence constraints; detail of the precedence constraint regulating robots 2 and 3 as they navigate through *C*_23_ is shown on the right.

In our evaluation, we use the multi-agent loosely-coupled coordination framework by Pecora et al. (2018); Mannucci et al. (2019) as a proxy for simulating the behaviour described in the previous section. The agents compute their motions to their current goal independently (i.e., without considering other agents), and their progress along the committed paths is supervised by a centralized coordinator which posts precedence constraints among pairs of agents to avoid collisions at intersections, as well as deadlocks and blockings. We have here extended the framework to include uncontrollable agents (e.g., pedestrians) by partitioning the intersections into two subsets: 1) robot-robot and robot-pedestrian intersections (henceforth called critical sections) where safety is ensured by the central coordinator, and 2) pedestrian-pedestrian intersections, where we assume the two interacting agent will locally coordinate by themselves. For each critical section, a precedence constraint will be imposed, defining which among the two agents should yield, where and until when (see also Figure 3).

#### 6.3.1 Definitions

Let R and H be the sets of robots and humans, respectively. With a small abuse of notation, the symbol □_*i*_ refers to variable □ of agent *i* in this section. Let *R*_*i*_(**x**_*i*_) be the collision space of the agent *i* when placed in a configuration **x**_*i*_ and $E_{i}\left(p_{i}\right)$ be the agent’s spatial envelope, that is, a polytope bounding the allowed spatial deviations while driving along the path **p**_*i*_ Andreasson et al., 2015). Consider a pair of agents (*i*, *j*) so as agent *i* or *j* is a robot with paths **p**_*i*_ and **p**_*j*_. Collisions may happen only in the set $S=\left\{\left(x_{i},x_{j}\right)\in X_{i}\times X_{j}|R_{i}\left(x_{i}\right)\cap E_{j}\left(p_{j}\right)\neq 0/\vee R_{j}\left(x_{j}\right)\cap E_{i}\left(p_{i}\right)\neq 0/\right\}$ . Let $C_{ij}$ be the decomposition of S into its largest contiguous subsets. Each set of configurations $C_{ij}\in C_{ij}$ is called a *critical section*. We parametrize each path **p**_*i*_ using the arch length *ζ*_*i*_ ∈ [0, 1] so as $p_{i}\left(0\right)=x_{i}^{\text{start}}$ and $p_{i}\left(1\right)\in X_{i}^{\text{goal}}$ . Furthermore, let *ℓ*_*i*_ ∈ [0, 1] be the arc length at which agent *i* first intersects *C*_*ij*_ along its path **p**_*i*_, that is, $p_{i}\left(\ell_{i}\right)=\inf_{p_{i}\;}C_{ij}$ .

New paths, the resulting critical sections, and precedences regulating access to them are computed/revised at a user-definable frequency 1/*T*_*c*_, while the fleet is in motion. The high-level control period *T*_*c*_ is typically chosen to be in the range [0.5, 2] seconds. The smaller the control period, the quicker agents react to progress of other agents (e.g., exhibiting less rubber-banding when queuing), and the more efficient the overall performance of the fleet is (Pecora et al., 2018).

Precedence orders are determined by user-provided heuristic function $h:{\left[0,1\right]}^{2}\times C_{ij}\mapsto \left\{0,1\right\}$ with *h*(*ζ*_*i*_, *ζ*_*j*_, *C*_*ij*_) = 1 indicating that $i\leq_{C_{ij}}j$ , i.e., agent *i* yields for agent *j* at the critical section *C*_*ij*_. A commonly used heuristic is:

$$h_{\text{dist}}\left(\zeta_{i},\zeta_{j},C_{ij}\right)=\left\{\begin{array}{cc} 1,\; & \;\text{if }\ell_{j}-\zeta_{j}\leq \ell_{i}-\zeta_{i}\\ 0,\; & \;\text{otherwise.} \end{array}\right.$$ (20)

The above heuristic realizes the “closest goes first” principle. This allows the agents to “follow” each other into critical sections, thereby avoiding the need to pre-define discrete areas of space for exclusive use of individual agents. Conservative models of the agents’ dynamics are used to exclude precedences which are not dynamically feasible; similarly, a model of the communication channel can be exploited to ensure safety under imperfect communication (Mannucci et al., 2019). Given a precedence order ${i<}_{C_{ij}}j$ , the precedence constraint $\left\langle p_{i},p_{j},{\bar{x}}_{i}\left(t\right),\sup_{p_{j}}C_{ij}\right\rangle$ is defined by computing the configuration ${\bar{x}}_{i}\left(t\right)$ at which the agent *i* is required to yield as:

$$\begin{array}{ll} & {\bar{x}}_{i}\left(t\right)=\left\{\begin{array}{cccc} & \max\left\{\ell_{i},r_{ij}\left(t\right)\right\}\; & & \text{if }\zeta_{j}\leq \underset{p_{j}}{\sup}C_{ij}\\ & 1 & & \text{otherwise} \end{array}\right.\\ & r_{ij}\left(t\right)=\underset{\zeta \in \left[\zeta_{i}\left(t_{i}\right),u_{i}^{C}\right]}{\sup}\left\{E_{i}^{\left[\zeta_{i}\left(t\right),\zeta \right]}\cap E_{j}^{\left[\zeta_{j}\left(t\right),\;\underset{p_{j}}{\sup}C_{ij}\right]}=0/\right\} \end{array}$$

where $E_{i}^{\left[a,b\right]}$ is the subportion of the envelope $E_{i}$ for *ζ*_*i*_ ∈ [*a*, *b*].

At the end each coordination loop, the closest constraint (called *critical point*) is updated and communicated to each agent. Safety holds indeed if all agents commit to stop in their current critical point (Pecora et al., 2018).

#### 6.3.2 Modeling humans

The motions of the two types of agents are computed as follows: the motions of robots in the set R follow the loosely-coupled approach (each path is computed by the robot’s planner and its trajectory obeys the precedence constraints imposed by the coordinator); the motions of humans in the set H are known (recorded trajectories) and hence replayed. A 2D simulation tool provided by the coordination framework (Pecora et al., 2019) is used to simulate the planned/recorded motions of robots/humans. Three possible test conditions—adamant human, adamant robot and cooperative agents—may be brought about by altering the heuristic *h* ∈ {*h*_h_, *h*_r_, *h*_dist_}, respectively.

In this paper, we use the cooperative agents heuristic. The cooperative agents setting is achieved by employing the *h*_dist_ heuristic shown in Eq. 20, reflecting the assumption that humans and robots cooperate to achieve the “fairest” outcome in giving precedence. This means that the agent closest to the critical section get precedence.

Although we do not use it in this paper, we also show the possible heuristic for the adamant human case for completeness. The adamant human heuristic gives a human precedence over a robot at critical section *C*_*ij*_ if the robot has not entered the critical section yet and the human is closer than a threshold distance *d* to critical section:

$$h_{\text{h}}\left(\zeta_{i},\zeta_{j},C_{ij}\right)=\left\{\begin{array}{cc} 1,\; & \text{if }i\in R,j\in H,\ell_{i}-\zeta_{i}\leq d\text{ and }\ell_{j}-\zeta_{j}\leq d\\ 0,\; & \text{if }i\in H,j\in R,\ell_{i}-\zeta_{i}\leq d\text{ and }\ell_{j}-\zeta_{j}\leq d\\ h_{\text{dist}}\left(\zeta_{i},\zeta_{j},C_{ij}\right),\; & \text{otherwise.} \end{array}\right.$$ (21)

The adamant robot heuristic *h*_r_ defines the opposite behavior, namely, humans yield to robots when both are in proximity of a critical section. By employing these heuristics in the coordination framework, we are able to replay the recorded trajectories of people alongside a robot executing its motions. Such a framework for simulation enables us to rapidly test multiple scenarios with several planners.

## 7 Description of experiments

In this section, we describe the parameters and data used in the different phases of our experiments.

### 7.1 Data and MoD-building

We use two different datasets: 1) simulated, and 2) real world data.

**FIGURE 4:**
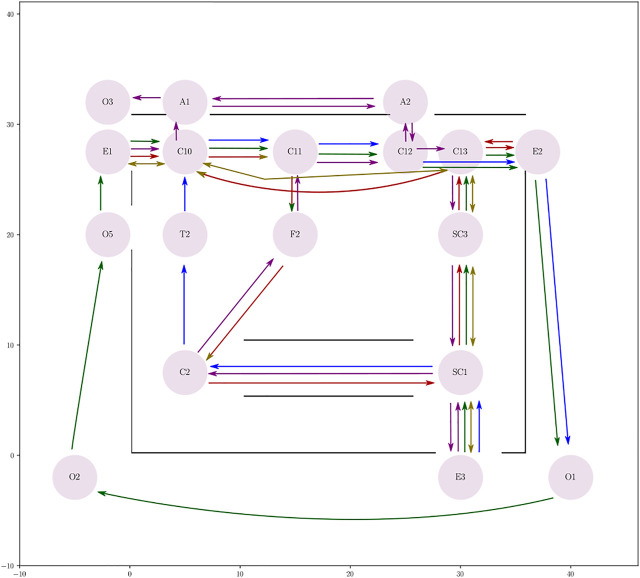
Set of waypoints and paths used to simulate workers in a warehouse-like environment using the PedSim simulator. Different colored arrows show the paths taken by different groups of pedestrians. Circles in light pink show the way points.

**FIGURE 5:**
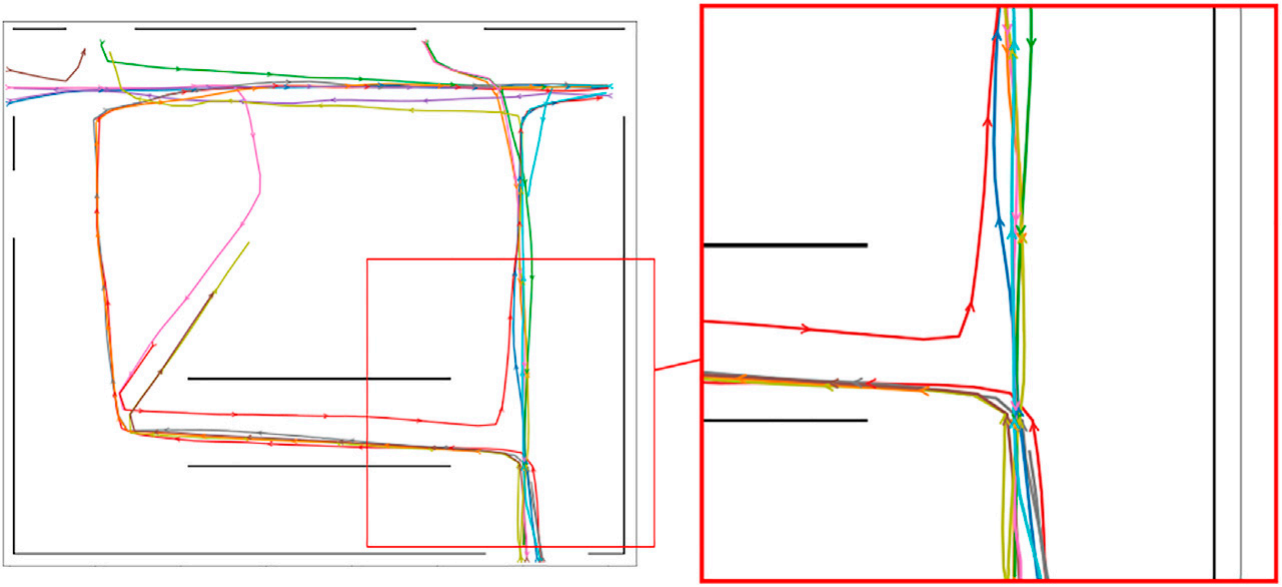
Pedestrian paths from time-point t1 used for execution phase experiments in the warehouse environment.

The first dataset is built using *PedSim* (Vasquez et al., 2014), a commonly used open-source pedestrian simulator. We simulate a set of workers moving in a warehouse-type environment. In this environment, people tend to follow well defined motion patters as described by the different colored paths in Figure 4. Most workers move through different stock areas of the warehouse to store pallet or pickup goods in the shelves area in the bottom (blue, purple, red). Other workers (managers) instead tend to move close to the walls. Using this simulation setup (i.e., definitions of pedestrian groups and way points, as shown in Figure 4), we have generated two separate datasets for MoD-building and testing by running the simulator twice while recording the pedestrian trajectories, as shown in Figure 5.

**FIGURE 6:**
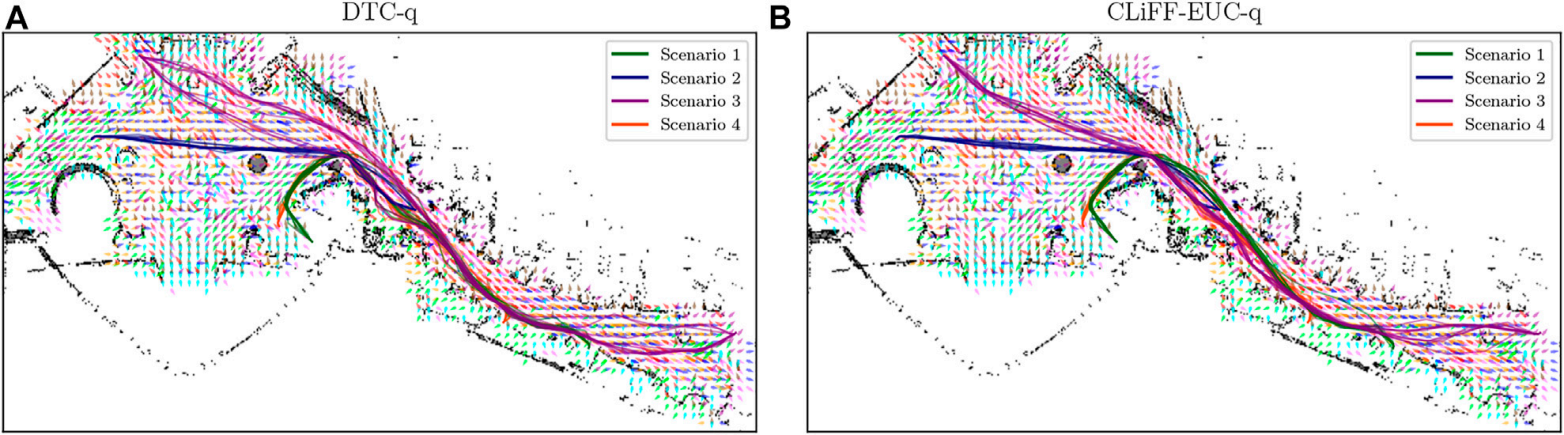
The CLiFF-maps and paths generated by the DTC-q **(A)** and CLiFF-EUC-q **(B)** planners in the ATC environment.

**FIGURE 7:**
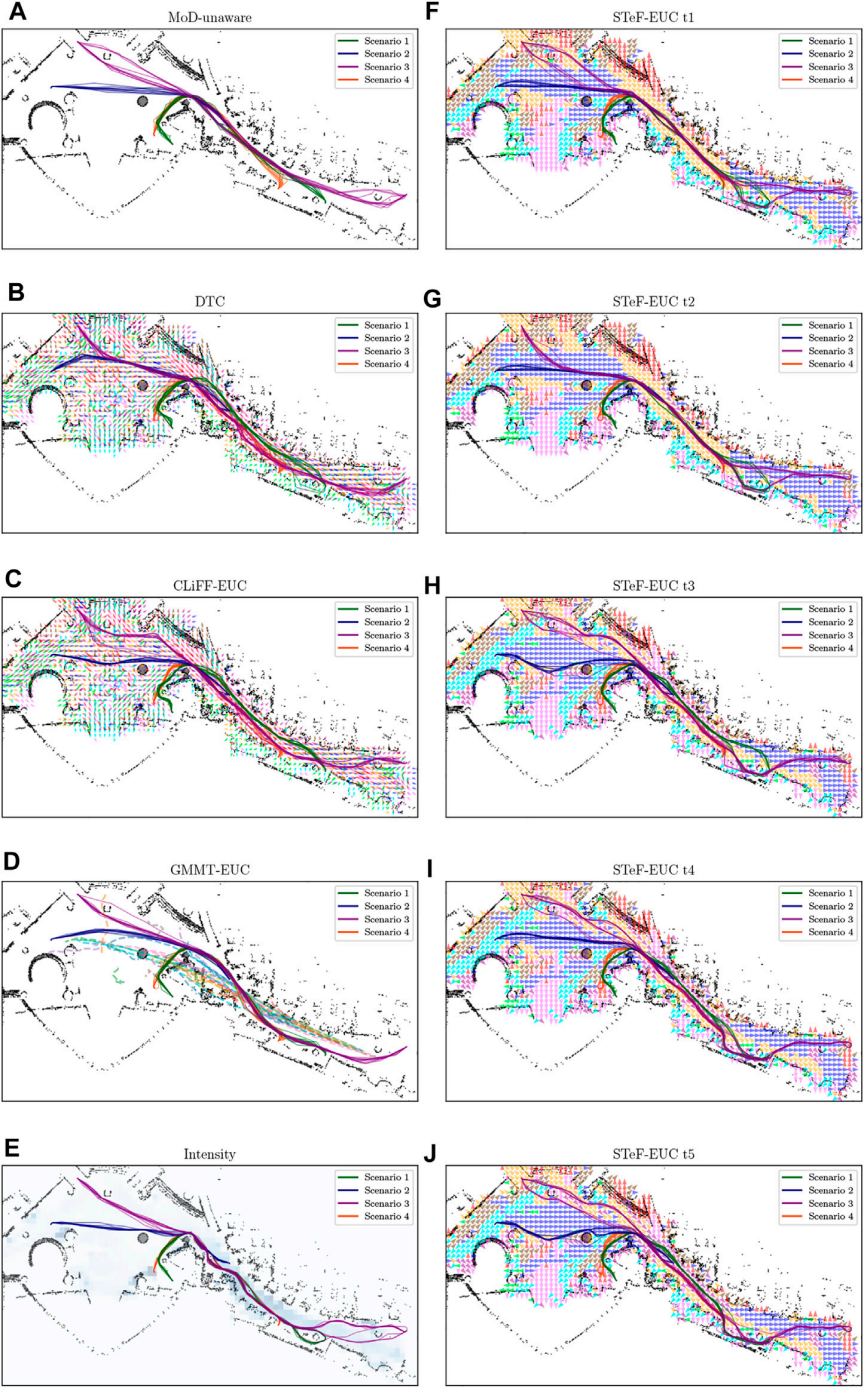
The different MoD maps and paths generated by the respective planners **(A–E)** in the ATC environment. The STeF-map plots **(F–J)** show arrows in each cell that corresponds to the discretized orientation with the highest predicted probability of motion at the respective time-points.

The second dataset comprises real pedestrian data from the *ATC* mall dataset [The Asia and Pacific Trade Center, Osaka, Japan, first described by (Brščić et al., 2013)]. This data set was collected with a system consisting of multiple 3D range sensors, covering an area of about 900 m^2^. The data has been collected between 24 October 2012 and 29 November 2013 every week on Wednesday and Sunday between 9:40 and 20:20, that gives a total of 92 days of observation. In the ATC dataset, we pick the first six consecutive days for training (MoD-building) and use the next 2 days for testing (execution phase experiments) as shown in Table 1. The resulting MoDs are shown (togheter with the executed paths) in Figure 6 (CLiFF) and Figure 7 (all).

**TABLE 1:** Days and dates from the ATC dataset used for training (MoD-building) and testing (execution experiments).

	Date	Day
Training	24 October 2012	Wednesday
	28 October 2012	Sunday
	31 October 2012	Wednesday
	04 November 2012	Sunday
	07 November 2012	Wednesday
	11 November 2012	Sunday
Testing	14 November 2012	Wednesday
	18 November 2012	Sunday

### 7.2 Planning phase

The setup of this phase consists of: 1) selecting the planning parameters such as planning duration, vehicle type, max vehicle velocity and path resolution, 2) choosing the appropriate MoD weight for each MoD’s cost function (see Section 4.2), and 3) selecting the set of start and goal locations for planning.

All our experiments consider a car-like vehicle and Reeds-Shepp paths as extend function (Reeds and Shepp, 1990) in line 7 of Algorithm 1. We chose a maximum vehicle velocity of $1\;\frac{m}{s}$ and a path resolution of 0.05 m for all planners in all environments. We also use uniform sampling of the state-space in line 5 of Algorithm 1 and a planning duration of 5 and 15 min in the PedSim and ATC experiments, respectively. We chose these values to give the planners enough time to find solutions that are as optimal as possible after a finite duration.

In Section 4.2, we saw how the weight *w*_*c*_ can limit the maximum path length for a zero-MoD-cost path. Suppose the path resolution is 0.05 m. This path contains $\frac{1}{0.05}=20$ points per meter. The worst case MoD-cost per meter (*μ*) is *μ* = 20*M*, where *M* is the worst case MoD-cost at a path/trajectory point. For DTC cost, *M* = 10, due to our limit on the maximum value of Mahalanobis distance. For the CLiFF-map EUC, *M* = 2, due to the maximum value of cosine distance. The values of *w*_*c*_ for each planner (see Table 2) are arrived at by setting the weighted worst case MoD-cost per meter (*w*_*c*_ *μ*) to 4 for all cost functions. In accordance with Section 4.2, *γ* = 1 + *w*_*c*_ *μ*. We choose a *γ* = 5, which means that if an initial solution is found to have this worst case MoD-cost (per metre), the planner would accept a zero-MoD-cost plan provided that its length is not greater than 5 times the length of the initial solution. Table 2 shows a list of all planners, their corresponding shorthands, along with their cost functions and MoD-weights.

**TABLE 2:** Table showing planner shorthands, MoD-type, the MoD-cost function used and chosen values of *w*_*c*_ for all planners used.

Planner shorthand	MoD type	MoD cost	MoD weight
MoD-unaware	(Figure 9)	(Figure 8)	0.00
DTC	CLiFF-map	DTC cost (Eq. 6)	0.02
CLiFF-EUC	CLiFF-map	CLiFF EUC (Eq. 9)	0.10
STeF-EUC	STeF-map	STeF EUC (Eq. 13)	0.10
GMMT-EUC	GMMT-map	GMMT EUC (Eq. 16)	0.10
Intensity	Intensity-map	Intensity Cost (Eq. 18)	0.20
DTC-q	CLiFF-map	DTC cost with q (Eq. 7)	0.02
CLiFF-EUC-q	CLiFF-map	CLiFF-EUC with q (Eq. 10)	0.10

**FIGURE 8:**
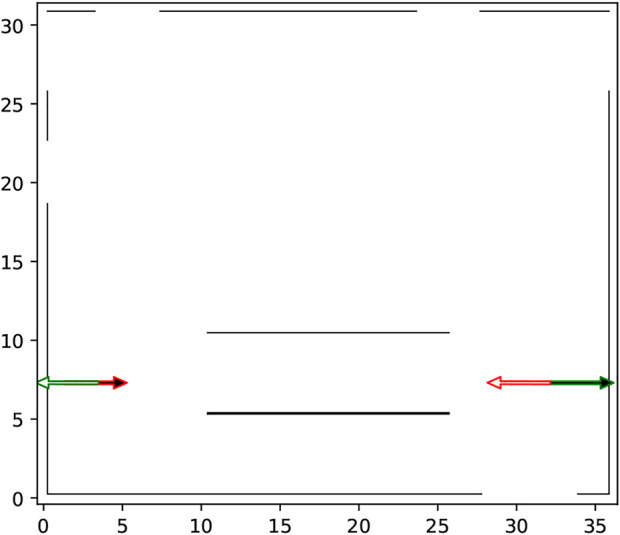
The occupancy map of the PedSim warehouse. The start and goal poses for the robot are outlined red and green, respectively. Both *x* and *y* axes are in meters.

Finally, we select different start and goal pairs Figures 6 and 8 and pedestrian reference trajectories. The related paths are displayed in Figures 5,10. For the PedSim experiments we choose two simple start-goal pairs. This setup is deliberately simple in order to emphasize the utility of MoDs to motion planning as we shall see in Section 8. For the ATC experiments, we select start-goal pairs such that all areas of the map are covered: one start-goal pair in the hall area (S2-G2), one spanning the entire map (S3-G3) and two that span both the hall and corridor areas partially (S1-G1 and S4-G4).

For STeF-map based planners we select five different time-points for planning, since STeF-map models temporal changes in motion patterns (see Section 5.2). Note that we also use the same time-points for the execution phase discussed in the next section. Due to the unavailability of real temporal data in PedSim (we would have had to manually add virtual temporal data), we do not use the STeF-map and the associated planner in the PedSim experiments.

### 7.3 Execution phase

In this phase we simulate the execution of the motion plans generated in the previous phase as explained in Section 6. For testing the execution of the generated motion plans, the testing part of the dataset. The pedestrian trajectories from the testing part is replayed as the robot motions are simulated. While simulating the robot using the coordination framework, we use a trapezoidal velocity profile with a maximum velocity of 1 m/s and a maximum acceleration of 1 m/s^2^. The coordination period is 1 s, i.e., the coordination framework computes/revises the critical sections and precedences every second.

### 7.4 Summary of experiments

**FIGURE 9:**
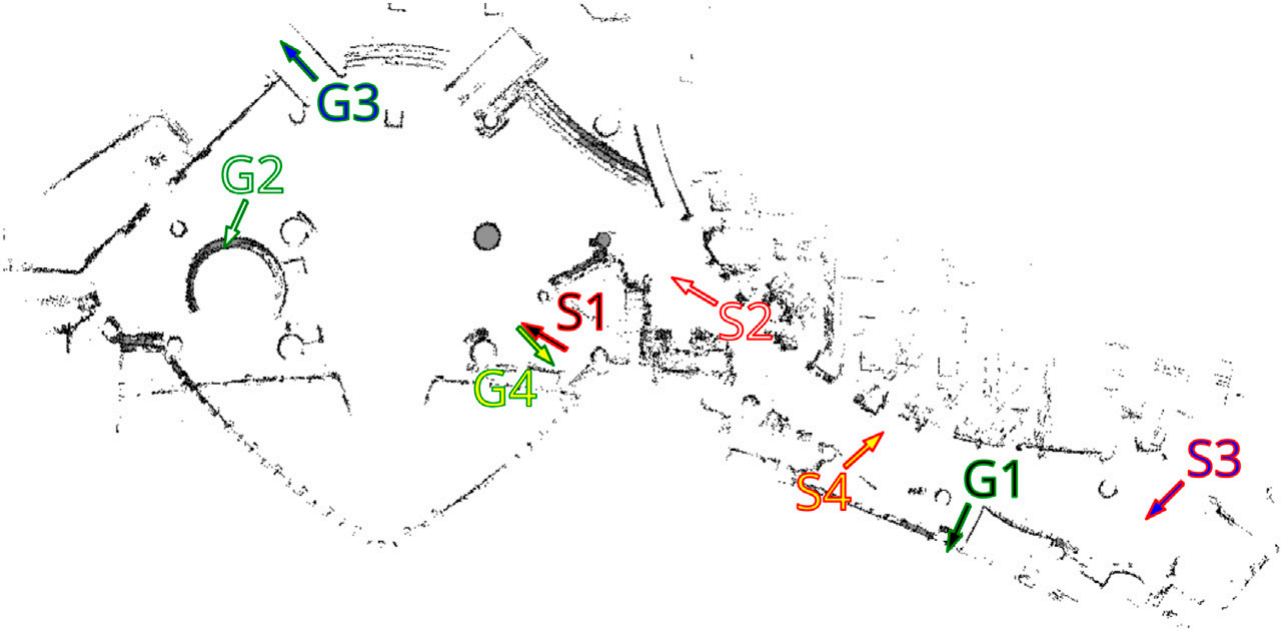
The occupancy map of the ATC shopping mall. The start and goal poses are outlined red and green, respectively.

Here we summarize the experiments in the PedSim and ATC environments (also shown in Table 3). For the ATC dataset, we use the first six available days for MoD-building and the next 2 days for execution-based benchmarking (Table 1). For PedSim, We generate two different datasets using the same setup (Figure 4). All our planners (Figure 2) use uniform sampling of the state space and the Reeds-Shepp vehicle type and its associated extend function. We use a path resolution of 0.05 m. In PedSim, each planner runs for 5 min, and in ATC, each planner runs for 15 min. We have defined two start-goal pairs for PedSim (Figure 8) and four for ATC (Figure 9). For the execution-based benchmarking, we cut-out 90 and 120 s starting at different time-points from the appropriate dataset. In ATC we choose five different time-points (from the 2 days marked for execution-based benchmarking). Similarly, in PedSim we choose six different time-points. Selected moments from the experiments in the ATC environment are shown in the video available at https://youtu.be/oUWOQENuHFc.

**TABLE 3:** Summary of experiments in the PedSim and ATC environments.

	Property	ATC environment	PedSim environment
1	Number of scenarios	4 (see Figure 8)	2 (see Figure 9)
2	Planning time-points	13:08:20, 18:00:00, 21:00:00 (14 November 2012), 14:46:40, 19:46:40 (18 November 2012)	six arbitrarily chosen durations from the testing dataset
3	Planning runs for STeF-EUC	10 runs per planning time per scenario	-
4	Planning runs for other planners	10 runs per scenario	10 runs per scenario
5	Planning duration	15 min	5 min
6	Total number of motion plans	400	100
7	Total number of execution phase experiments	1,200	600
8	Total time taken for all experiments	140 h	40 h

## 8 Results

In this section we look at the results from the planning and execution phases of the experiments. As discussed in the previous section, we perform these experiments in two environments, PedSim and ATC, using the corresponding datasets. First we look at the planning phase results that we use to motivate the need for experiments based on execution. Next we present the execution phase results from the PedSim environment and reason about them using the motion plans. It is possible to explain results in the (simple) PedSim scenarios using the motion plans, to some extent. Next, we present the results from the ATC dataset that are harder to explain by simply looking at the motion plans.

### 8.1 Planning phase results

We now look at traditional metrics used in conjuction with motion plans. Specifically, we look at graphs showing different metrics based on the motion plans themselves.

Figure 11 shows boxplots of Euclidean distance cost (*c*_*d*_), quaternion distance cost (*c*_*q*_) and weighted MoD cost (*w*_*c*_ *c*_*c*_) from the pedsim warehouse (a)–(c) and ATC (d)–(f). Note that as mentioned in Section 7.2, we do not use the STeF-map and the associated planner in the PedSim experiments because of the lack of real temporal data in PedSim. As expected, the MoD-unaware planner results in the least Euclidean and quaternion distance cost. Obviously, MoD-cost is undefined for MoD-unaware planner, which does not consider MoD-information. In the ATC environment, Figure 11D–F, GMMT-EUC’s quaternion distance cost overlaps with that of the MoD-unaware planner in all cases, whereas CLiFF-EUC, DTC, STeF-EUC and Intensity have comparably higher quaternion distance cost. The GMMT-EUC cost does not change as much locally as DTC, CLiFF-EUC, STeF-EUC and Intensity costs, and therefore, the turns are fewer (see Figure 6). This is due to the nature of the maps and their cost functions, i.e., GMMT-map consists of motion patterns represented as a trajectories and the cost function is weighted by the standard deviation as seen in Eq. 16. The effect is that, GMMT-EUC-cost is zero in regions outside one standard deviation of the distributions defined in the GMMT-map. However, the other MoD-costs are non-zero for all regions where observations (pedestrians) exists. Because of this, the MoD-cost is also lower for GMMT-EUC compared to other MoD-aware planners.

**FIGURE 10:**
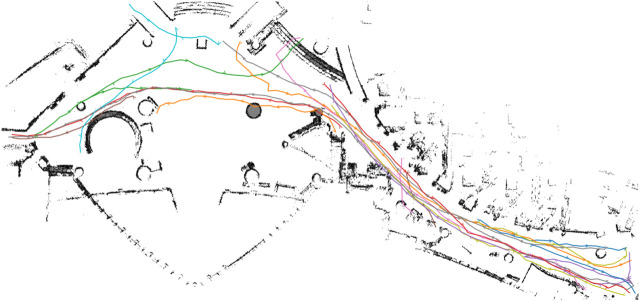
Pedestrian paths from time-point t1 used for execution phase experiments in the ATC environment.

**FIGURE 11:**
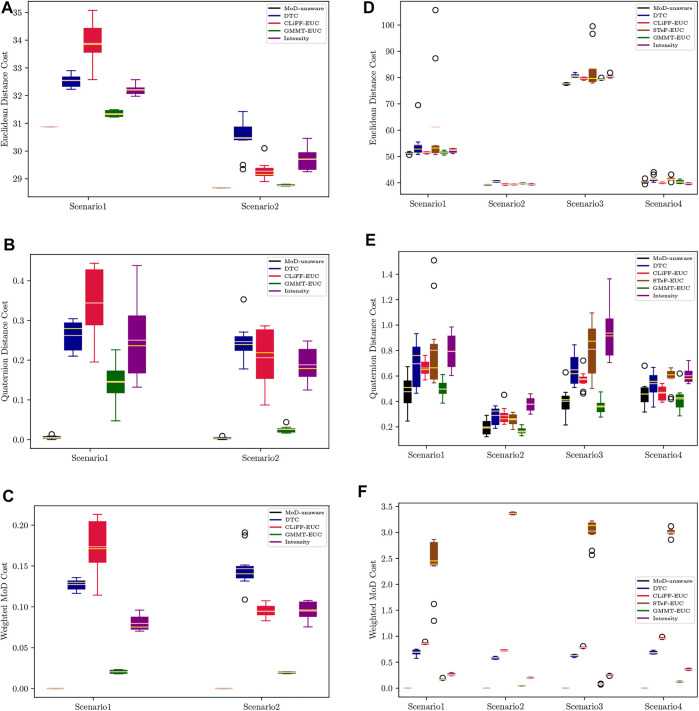
Planning phase stats from the pedsim warehouse **(A–C)** and ATC **(D–F)** respectively. First row **(A,D)** is the Euclidean distance cost, second row **(B,E)** is Quaternion distance cost and third row **(C,F)** is weighted MoD cost per meter. Means are shown in pink and medians in yellow.

However, these metrics do not provide an indication as to which cost function would lead to the best execution efficiency. As we have mentioned before, the only connection between the MoDs is the data that is used to generate them and the choice of weights. Recall that we have chosen the weights such that Next, we look at the execution phase results.

### 8.2 Execution phase results

First we look at the results from the PedSim warehouse environment. This setup is deliberately simple in order to emphasize the utility of MoDs to motion planning. Specifically, in this case, it is easier to reason why MoD-aware planners are better, and which MoD-aware planners are better, by looking at the generated paths and the objective measures. However, in the ATC environment, it is harder to judge the utility of MoDs by looking only at the generated motion plans.

#### 8.2.1 PedSim warehouse

**FIGURE 12:**
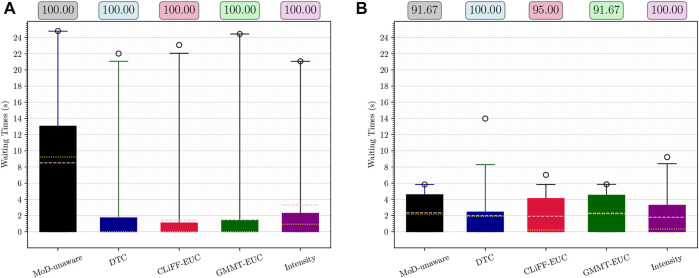
Waiting times and success rate (on top) for Scenario 1 **(A)** and Scenario 2 **(B)** from PedSim warehouse environment. Means are shown in pink and medians in yellow.

**FIGURE 13:**
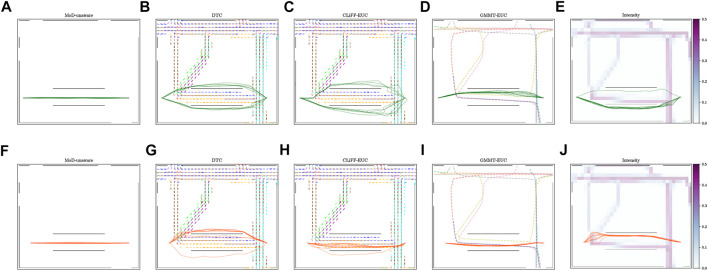
Paths generated by different planners and related MoD map for Scenarios 1 **(A–E)** and 2 **(F–J)** in the PedSim warehouse environment. Column-wise from left to right: MoD-unaware planner, DTC, CLiFF-EUC, GMMT-EUC, Intensity.

Figure 12 shows the waiting times using boxplots with the corresponding success percentage (as defined in Section 6.2) shown on top of each boxplot. Figure 13 shows the paths and MoD-maps for the PedSim warehouse environment. We have two simple scenarios as shown in Figure 8: Scenario 1, where the robot goes from right to left, and Scenario 2, where the robot goes from left to right. The corridor in the bottom has two distinct flows as can be seen from the CLiFF-map in Figure 13B: blue arrows in the top half of the corridor indicate motion is towards the right, and yellow arrows in the bottom half of the corridor indicate that the motion is to the left.

In the first scenario (moving from right to left in Figure 8), the CLiFF-EUC, the DTC and the Intensity planners tend to avoid the crowded area (i.e., corridor) entirely. While the CLiFF and GMMT planners do not explicitly include information about the intensity of the flow, given the cost functions in Eqs 9, 16, they implicitly account for intensity in this scenario, since there are no distributions where there has been no flow observed. The time wasted is consequently very low for MoD-aware planners (median below one second) whereas the MoD-unaware planner wastes 9 seconds (median).

In the second scenario (moving from left to right in Figure 8), the DTC planner and Intensity planner avoid the flow entirely, whereas the CLiFF-EUC planner and GMMT-EUC planner try to follow the flow within the corridor. Although the time wasted is comparable to that of other planners, the success rate of the planners (that is, where robot motions can be completed without replanning as seen in Section 6.2) is higher for DTC and intensity planners. Judging by the results in Figures 12, 13, it is better to avoid the flow altogether in this scenario, rather than attempting to enter and follow it, which means that intensity information is more important to the motion planner than information about direction.

Although CLiFF-EUC and GMMT-EUC account for intensity implicitly, their costs are zero (by design) when the robot’s velocity vector is aligned with the direction modelled in the MoDs. Since DTC-cost is based on the Mahalanobis distance, the velocity vector should match the underlying distribution, otherwise the cost is high. This means that, if the flow of pedestrians has very low spread (as they do in our PedSim setup), DTC-cost along these regions is likely to be very high (due to the low covariance especially in direction). This is why DTC avoids the corridor area entirely. In summary, planners that avoid the flow of people altogether have higher success rates than planners that enter the corridor.

In the case of these experiments with simulated data, it was possible (to some extent) to gauge the quality of plans based on the plots. That is, planners that avoided the corridor area, produced better results during execution. However, in the case of the ATC environment, it becomes increasingly difficult to reason about the plans (see Figure 6). Also, comparing the planning phase results from PedSim environment (Figure 11) to the execution phase results (Figure 12, it is clear that planning phase metrics alone are not a good indicator of the quality of the paths. The MoD-unaware planner has the lowest distance costs, but inevitably leads to difficulties when executing motions alongside other agents (pedestrians). Therefore, objective metrics from execution of motion plans is important.

#### 8.2.2 ATC dataset

**FIGURE 14:**
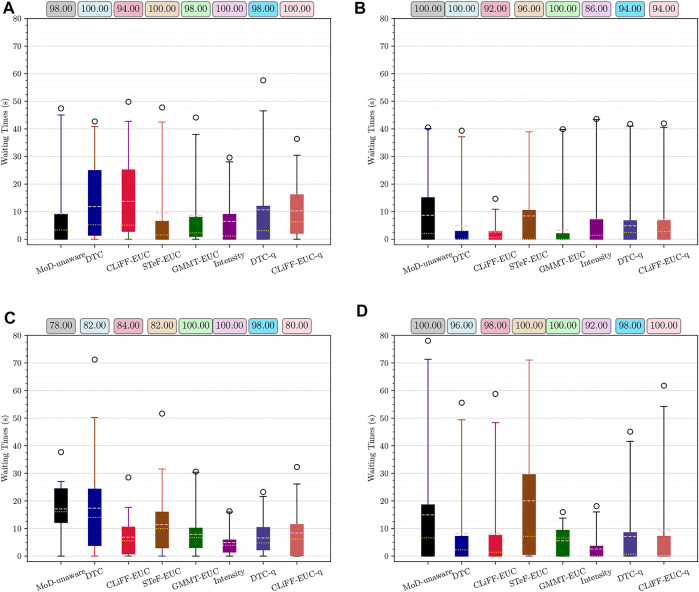
Waiting time and success rate for Scenario 1–4 **(A–D)** respectively, from the ATC environment. Means are shown in pink and medians in yellow. Whiskers extend from 0 to 99%.

For the experiments with real-world data from the ATC data set, we begin our analysis by presenting graphs of the waiting times for each planner. Figure 14 shows waiting times for each planner categorized by the scenario. For instance, Figure 14A shows the waiting times due to execution of paths from each planner for scenario 1, at all time-points. Similarly, Figure 14 shows the results categorized by the planning time-point. For example, Figure 14A shows the waiting times for each planner from all scenarios, at time-point 1. Finally, Figure 16 shows the different MoD maps and paths generated by the respective planners in the ATC environment.

**FIGURE 15:**
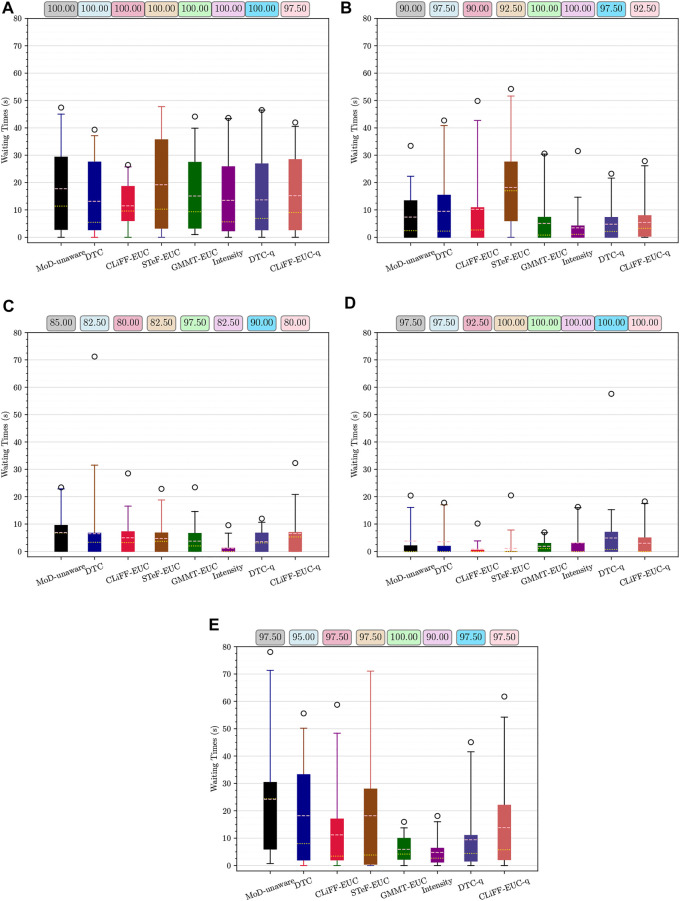
Waiting time and success rate for time-points 1–5 **(A–E)** respectively, from the ATC environment. Means are shown in pink and medians in yellow. Whiskers extend from 0 to 99%.

**FIGURE 16:**
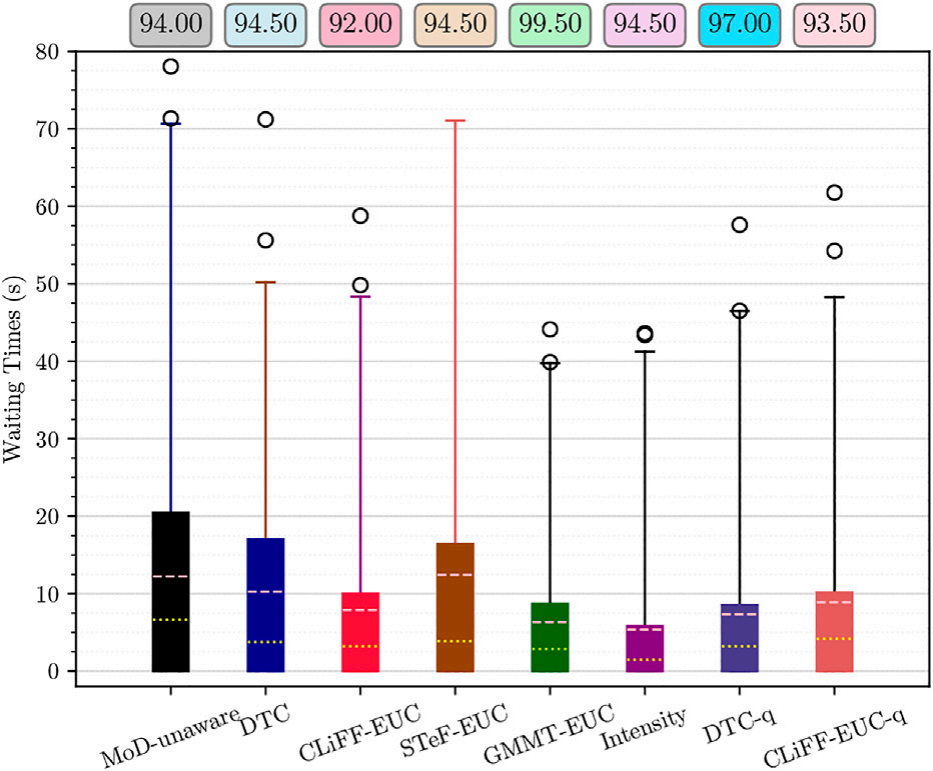
Waiting time and success rate all scenarios and all time-points. Means are shown in pink and medians in yellow. Whiskers extend from 0 to 99%.

It is clear from the overall boxplot (Figure 15F) that MoD planners lead to less waiting time (both mean and median) compared to the MoD-unaware planner. Moreover, only the CLiFF-EUC planner has a smaller success rate compared to MoD-unaware planner. In summary, we make the following observations: 1) GMMT-EUC gives the best results when executing plans in the ATC scenarios. It has nearly 100% success rate, and the mean waiting time of ∼6 s compared to a mean waiting time of ∼12 s for the MoD-unaware planner after 200 executions (5 time-points × 4 scenarios × 10 plans). 2) Intensity has the shortest waiting times (mean and median) but suffers from lower success rate. 3) Only STeF-EUC produces higher mean waiting time compared to the MoD-unaware planner. But, it too has a higher success rate compared to MoD-unaware planner.

Next, we break down these results first by scenario and then by time-point.

##### 8.2.2.1 Scenario-wise analysis

In Scenario 2, the robot starts at end of the long corridor area and passes through the large hall area. Since there is a larger area for the planner to find low-cost solutions, all MoD-aware plans waste less time compared to the MoD-unaware planner. In particular, the DTC planner and the GMMT-EUC planner have 100% successful execution and zero median time wasted (see Figure 14B). The full CLiFF-map planners (DTC-q and CLiFF-EUC-q) have similar waiting times to the Intensity planner, but higher success rate than Intensity planner. When comparing to their “non-q” counterparts, the use of *q* value improves the success rate of CLiFF-EUC by 2% while the success rate of DTC drops by 6%. In this scenario, planners utilizing direction explicitly (GMMT-EUC, CLiFF-EUC and DTC planners) have low waiting times.

The robot has to enter the corridor from the hall area in Scenario 1 and has to leave the corridor in Scenario 4. The MoD-unaware planner wastes more time while leaving the corridor (Scenario 4) than while entering (Scenario 1). In Scenario 1 (entering the corridor), DTC and CLiFF-EUC waste more time than MoD-unaware planner on average. This is reduced when utilizing the motion ratio *q* (DTC-q and CLiFF-EUC-q). In Scenario 4, DTC-q and CLiFF-EUC-q have similar waiting times to their “non-q” counterparts, but have 2% better success rate. However, GMMT-EUC and Intensity seem to perform similarly in both situations. Intensity information seems most relevant while entering/leaving the corridor since both the GMMT-EUC planner and the Intensity planner have very low spread in time wasted.

Scenario 3 involves the longest distance between start and goal. Consequently, the time saved by MoD-aware planners is most pronounced in this case. Intensity (explicit intensity information) and CLiFF-EUC and DTC (explicit direction) planners have lower mean and median waiting times and higher success rate than MoD-unaware planner. The time wasted is also considerably smaller for MoD-aware planners compared to the MoD-unaware planner, except DTC planner. DTC-q has much higher success rate and lower waiting times compared to its “non-q” counterpart. The GMMT planner (utilizes direction explicitly and intensity implicitly) has the best results in Scenario 3. DTC-q planner (utilizes both direction and intensity explicitly) has slightly lower mean waiting time compared to GMMT, but also 2% lower success rate.

##### 8.2.2.2 Crowdedness

In order to understand what testing time-points are more crowded than others, we use Fundamental Diagram (FD) of flow. FDs represent the relationship between pedestrian velocity and crowdedness. Duives et al. (2015) provide an overview of different types of fundamental diagrams. In this paper, we use X-T plots (Edie (1963) as cited by Ni and Leonard (2006)).

**FIGURE 17:**
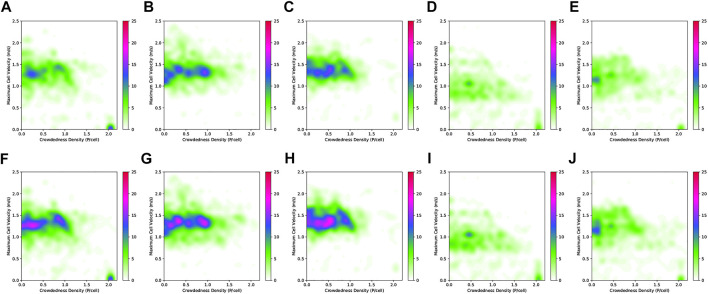
Histograms of Fundamental Diagrams of flow of the long corridor **(A–E)** and hall **(F–J)** areas of the ATC environment. Column-wise from left to right: time-points 1–5.

In X-T plots, the space is divided into uniform square cells. In addition, the time domain is also split into regular time periods. Crowdedness is computed at each cell (*x*, *y*, *t*) using the time spent by each unique pedestrian in the particular cell within the particular time period. Essentially, crowdedness is computed using several crowdedness maps—one grid-map per time period. Additionally, a velocity value is associated to each cell at all time periods—the maximum velocity experienced at the cell. By plotting the crowdedness versus the velocity, we obtain the fundamental diagram. Finally, a histogram is created from the fundamental diagrams. Figure 17 shows the fundamental diagrams (2d-histograms) computed using X-T maps, of the corridor and hall areas. Dense peaks in the histogram plot of the FDs, such as those seen in first three columns of Figure 17, mean that the same region has or different regions have the corresponding velocity and crowdedness values at various time periods. Conversely, in the last two columns of Figure 17, the velocity and crowdedness values are spread out, i.e., at various time periods, the crowdedness and velocity values vary a lot.

##### 8.2.2.3 Planning-time-point-wise analysis

When looking at the results classified based on time-point of planning (Figure 15) in conjunction with the crowdedness maps, a few inferences can be made: 1) Success rate of the planners is greater in time-points 4 and 5 compared to 1, 2 and 3. 2) Median time wasted by all MoD-aware planners is lower than that of MoD-unaware planner in time-point 3, 4 and 5. 3) Fundamental diagrams for time-points 2 and 3 show dense peaks (at higher crowdedness values) compared to 4 and 5. This means that the velocity and crowdedness are both spread out more in the latter case.

It can be seen from the fundamental diagram of time-point 1 that some pedestrians slow down or stop (dense peak at zero velocity and high crowdedness). This is possibly why the MoD-aware planners do not waste less time. When crowdedness and velocity are more spread out (time-points 4, 5) the MoD-aware planners have better success rate and lower time wasted than the MoD-unaware planner. When crowdedness and velocity show dense peaks (time-points 1–3), the difference between the planners in terms of time wasted is less pronounced. This is probably because MoD-awareness does not benefit the planners as much at these levels of crowdedness as it does at lower levels of crowdedness. However, the median time wasted and success rate are both better for at least one MoD-aware planner in all cases. Especially, GMMT-EUC has the best success rate at time-points 1–3. DTC-q performs similarly to GMMT-EUC at all time-points, while having slightly lower success rate.

A major difference in the MoD-maps from the ATC dataset, compared to the PedSim warehouse is that the MoD-maps are much denser. Simulated pedestrians in the PedSim warehouse follow specific paths with little variability in the paths. This results in the MoDs being sparse. That is, for example, CLiFF-map distributions are absent where no motion was observed and some intensity-map values will read zero. Therefore all planners in the PedSim warehouse environment will implicitly account for intensity information. However, in the ATC environment, besides Intensity-map, only the GMMT-map accounts for intensity information (implicitly, due to the nature of the GMMT model). Notice that although the CLiFF-map model is capable of accounting for intensity *via* *q*, the motion ratio, we have not used it in the cost function in order to better discriminate the effect of different kinds of dynamics information.

The CLiFF-q and DTC-q planners have the same or better success rate compared to their “non-q” counterparts at all time-points.

##### 8.2.2.4 Summary

The results from the ATC environment can be summarized as follows:

• In the hall regions (Scenario 2), planners utilizing direction information have better waiting times and success rate.
• While entering and leaving the corridor (Scenario 1 and 4), planners utilizing intensity information have better waiting times and success rate.
• When there is a combination of hall and corridor areas, planners utilizing intensity (Intensity and GMMT-EUC planners) have better waiting times and success rate.
• When crowdedness values are more spread out (from the FDs), the MoD-aware planners show considerably better results (compared to the MoD-unaware planner) than when the crowdedness values are concentrated.
• Overall, the GMMT-EUC planner followed by DTC-q have the best results in terms of time wasted during execution of motions (waiting time) and percentage of executions completed without replanning (success rate).

## 9 Discussion

### 9.1 Possible avenues for future work

In this paper we have proposed objective metrics to evaluate the utility of MoDs to global motion planning. In this section, we discuss possible avenues for future research and development.

With regards to the benchmarking framework, the experiment ends if the robot needs to replan. The multi-agent coordination framework currently provides the ability to modify motion plans both at the global level (*via* calls to the motion planner) and at the local level (by leveraging the concept of spatial envelopes to allow bounded deviations from the nominal path). However, a fast replanning method is lacking. This entails that robots can potentially re-plan, but only by calling the global planner, which may increase the waiting time unfairly. If fast replanning, for example, TEB planner by Keller et al. (2014), is indeed available, it is possible that a robot does not waste several seconds waiting for an agent while it computes a new plan. This means that all experiments would potentially succeed and one could count the number of times replanning was necessary instead of the success rate.

Another possible improvement to the simulation framework is to enable replanning for pedestrians/uncontrollable agents. This makes it possible for a pedestrian to navigate around a robot that might be stuck waiting for the pedestrian or vice versa. Also if replanning is possible for agents and the robot, although the total time wasted could be large, the mission can be finished.

To evaluate the practical utility of using MoDs for motion planning, we have proposed a simulation framework which includes real-world trajectories to simulate human motions. This choice aims to allow repeatability of experiments. Real-world experiments are indeed hard to reproduce, besides the difficulty in finding enough participants and ensuring safety standards. However, performance in terms of human-robot interaction (e.g., the level of robot acceptability and perceived safety) still requires real-world data due to the low fidelity of current simulators, and these metrics are equally important for robots operating in human-populated environments. To this end, we plan to extend our real-world evaluation to include subjective metrics (such as questionnaires about acceptability) to investigate the impact of MoDs-aware planners on perceived safety.

We have tested different cost functions using the RRT* planner in a simulation framework. In real experiments, where planning duration is more important, it might be advantageous to consider other planners such as space-lattice planners, anytime A*, etc. If, on the other hand, we continue to use sampling-based motion planners, we would need sophisticated sampling techniques so as to reduce the planning duration required to obtain low-cost solutions. In this paper, in order to focus on the objective quality of the generated motion plans, we have given the planners an arbitrarily large enough amount of time to generate motion plans. When planning in real-time, more informed sampling functions would be necessary for the generation of motion plans (and re-plans) in a reasonable amount of time.

### 9.2 Conclusion

Previous research in the area of human-aware motion planning and motion planning in dynamic environments has focused heavily on live dynamics information. Also, oftentimes, it is assumed that the entirety of the environment is visible. The use of MoDs (maps of dynamics) entail that a robot can plan human-aware motions also in regions that it cannot currently observe. For instance, even a simple MoD such as the Intensity map can help robots be more efficient (as seen from the results in Figure 16) without the need for additional sensors in the environment.

We have discussed how to incorporate an additional MoD-cost to the cost function typically used in motion planning. We have also presented three new cost functions, for STeF-map, GMMT-map and Intensity-map, and proposed minor changes to existing cost functions for CLiFF-map. The cost function and their respective MoDs emphasize different aspects of the dynamics information such as intensity, direction, speed, etc., that can be relevant for different applications. Based on the application, one might choose an appropriate MoD and cost function. For instance, when avoiding crowds is a lot more important than following the flow, one might use the Intensity-map alone. When both are important, one might use the CLiFF-map with the corresponding cost function that includes intensity information. When the flow of people is very strict (such as in the simulated environment in Figure 5), the GMMT-map may be used.

We have tried to address the problem of assessing and quantifying the utility of incorporating environmental dynamics information available as MoDs in motion planning. Our contribution in this regard is that we have motivated the need for execution-based benchmarking by pointing out that traditional metrics alone are not enough to gauge the benefits of MoD-awareness in a motion planner (Sections 8.1 and 8.2.1). Besides, we have designed novel metrics that objectively gauge the utility of MoDs. These metrics measure the efficiency of a robot and the disruption it causes. By measuring the time wasted by a robot and the percentage of successful executions, we quantify the efficiency of the robot. By measuring the time wasted by the pedestrians we quantify the disruption caused by the robot. These metrics are not only applicable to gauge the utility of MoDs in motion planning, but also the utility of other types of dynamic information. For instance, these metrics may also be employed to evaluate the utility of a motion prediction in motion planning for a robot.

We have presented a benchmarking method involving these objective metrics for conducting evaluations (Section 6). This contribution is novel in the sense that it is the first work to evaluate the utility of MoDs in motion planning that is based on *execution* of motion plans and not merely on the motion plans themselves.

We have motivated the need of a simulation framework capable of reproducing real-world experiments in Section 1. The simulation framework we propose allows us to reuse existing pedestrian datasets, thereby reducing the data collection effort that might otherwise be necessary. Besides, a framework based on simulation entails that benchmarking is easily reproducible and can be done rapidly compared to testing in the real world. Just as the metrics, the simulation framework is not exclusive to testing the utility of MoDs: it is equally applicable in evaluating the utility of both MoDs and live dynamics information.

We have also conducted and presented a thorough comparison of motion planners that use the different MoD-aware cost functions (Section 8.2). The results show that cost functions that account for both direction and intensity information produce the most efficient (least time wasted) motion plans. These results suggest that MoD information benefits the efficiency of execution of paths. Furthermore, the results helps further development of both MoDs and their cost functions with respect to their utility to motion planning.

## Data Availability

The datasets for performing the study and links to all software and instructions for performing the experiments in this paper are available at https://ksatyaki.github.io.

## Footnotes

1 The interested reader is referred to the work by Kucner et al. (2020b) for a detailed overview.
